# Causal Associations Between Cystatin and Lung Cancer: A Two‐Sample Mendelian Randomization Study

**DOI:** 10.1111/crj.70112

**Published:** 2025-07-11

**Authors:** Chunling Zhang, Riya Wu, Hang Liu, Shihuan Yu

**Affiliations:** ^1^ Department of Pulmonary Disease The First Affiliated Hospital of Harbin Medical University Harbin Heilongjiang China

**Keywords:** causal correlation, cystatin, lung adenocarcinoma, Mendelian randomization, non‐small cell lung cancer, squamous cell lung carcinoma

## Abstract

**Introduction:**

The cystatin family is particularly relevant in lung cancer research due to its links to inflammation, protease balance, and tumor progression. Although population‐based studies have documented associations between cystatin and lung cancer, causal relationships remain undetermined.

**Methods:**

Based on genomic statistics of seven different cystatins and three subtypes of lung cancer, we conducted a two‐sample Mendelian randomization (MR) study. The inverse‐variance weighted (IVW) method was the main approach for causality estimation. The weighted median, simple mode, weighted mode, and MR‐Egger regression methods were further employed to validate the main findings. In the sensitivity analysis, horizontal pleiotropy was assessed by MR‐Egger regression and Cochran’s Q test. MR‐PRESSO and Radial MR methods were used to identify heterogeneity and remove outliers.

**Results:**

Genetically predicted Cystatin 8 was causally associated with squamous cell lung carcinoma (OR = 1.062, 95% CI: 1.004–1.124, *p* = 0.035). No causal relationships were found for genetically predicted cystatin 8, ‐B, ‐D, ‐F, or ‐M with squamous cell lung carcinoma, lung adenocarcinoma, and NSCLC. However, outliers were identified between Cystatin D, ‐M, and ‐F using MR‐PRESSO and Radial MR. After the removal of outliers, the association between Cystatin D and lung adenocarcinoma turned significant (OR = 1.178, 95% CI: 1.023–1.358, *p* = 0.023). Sensitivity analyses confirmed the robustness of main results after outliers removal.

**Conclusion:**

Genetically predicted Cystatin 8 was causally associated with squamous cell lung carcinoma. Future population‐based studies are required to substantiate these results.

## Graphical abstract

This Mendelian randomization study revealed a causal association between genetically predicted cystatin 8 and squamous cell lung carcinoma, while Cystatin D was linked to lung adenocarcinoma after outlier adjustment. The findings suggest cystatins may play distinct roles in lung cancer subtypes, calling for further investigation to confirm these relationships.

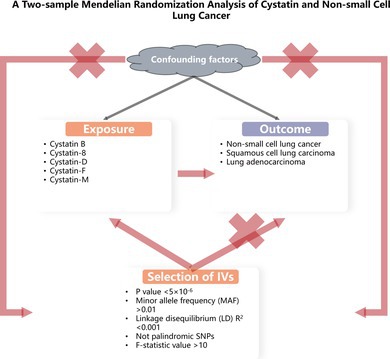

## 1 Introduction

Lung cancer remains the primary cause of cancer‐related mortality worldwide, encompassing two major types: non‐small cell lung cancer (NSCLC) and small cell lung cancer (SCLC). Among these, NSCLC is the most common, with adenocarcinoma and squamous cell carcinoma being the predominant histological subtypes [1]. Squamous cell lung carcinoma originates in the central bronchi, which is strongly associated with smoking [2]. Lung adenocarcinoma, on the other hand, typically develops in the peripheral lung tissues [3]. The incidence of lung cancer is still at a high status, which has exerted heavy burdens on public health. In 2020, lung cancer accounted for 2.2 million new diagnoses and 1.8 million deaths globally [4]. Lung cancer patients often present with advanced‐stage disease due to the asymptomatic nature of early‐stage tumors and the lack of effective screening programs. Despite advances in treatment, the prognosis remains suboptimal, with a 5‐year survival rate of only 15% for NSCLC patients [5]. Reduced quality of life is frequently reported by lung cancer patients due to symptoms such as chronic cough, dyspnea, chest pain, and systemic effects like weight loss and fatigue [6]. Understanding the pathogenesis and identifying potential therapeutic targets for lung cancer are critical research priorities.

Recent studies have highlighted the role of cystatin in cancer biology [7]. Cystatins comprise cysteine protease inhibitors, which may contribute to several pathological processes, including cancer [8]. For instance, Cystatin 8 is primarily involved in reproductive biology but may also contribute to cancer pathogenesis [9]. Cystatin D modulates immune responses and inflammation, key processes in tumor development [10]. Cystatin F exerts a regulatory effect on cytotoxic T lymphocytes and natural killer cells, which protect against tumors via immune surveillance [11]. Population‐based evidence revealed the positive correlations between serum cystatin C levels and mortality from lung cancer [12]. Besides, cystatin SN was positively associated with recurrence and metastasis rates in NSCLC cases [13]. To date, the causality between cystatins and lung cancer has not been definitively determined. Observational studies are often confounded by confounding factors and reverse causation, making it challenging to infer a direct causal relationship.

To address these limitations, Mendelian randomization (MR) analysis has emerged as a robust method for evaluating causality, using genetic variants as instrumental variables (IVs) [14]. This approach capitalizes on the random allocation of alleles during meiosis and effectively simulates the randomization seen in clinical trials, thereby mitigating confounding and reverse causation [15]. The validity of MR analysis relies on three key assumptions: genetic variants must be strongly associated with the exposure, remain independent of confounding variables, and affect the outcome only through the exposure [16]. A two‐sample MR study was conducted to investigate the causal link between cystatin levels and lung cancer risk. This research is aimed at elucidating the role of cystatin in lung cancer etiology, potentially paving the way for biomarker‐based strategies in the management and treatment.

## 2 Methods

### 2.1 Study Design

**FIGURE 1:**
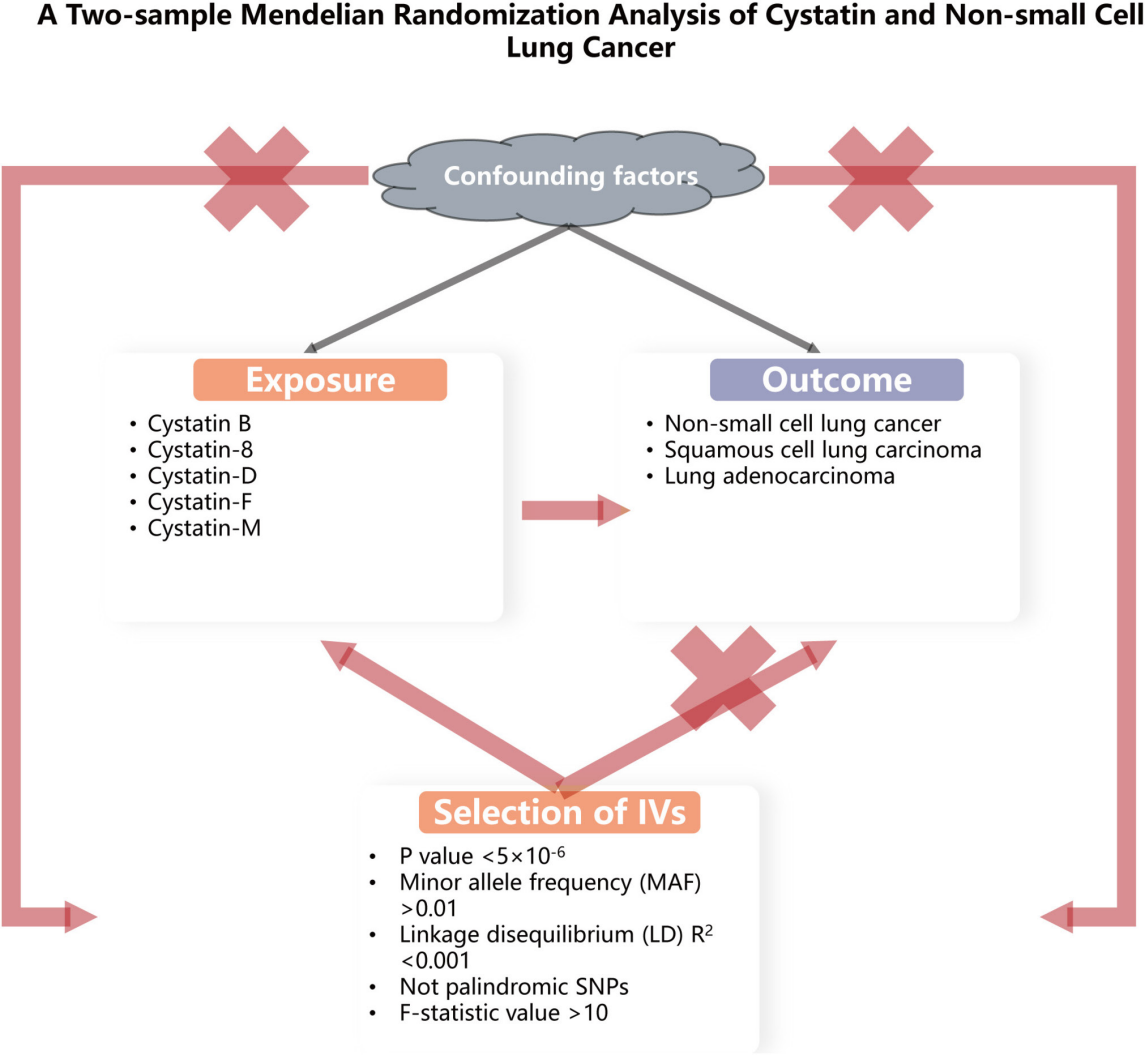
Flowchart of the study design.

Causal relationships between cystatin and lung cancer were explored based on a two‐sample MR study. Using data from previously published studies with obtained consent, genetic variants associated with cystatin were collected. IVs were selected based on three core assumptions [16]: (A) The single nucleotide polymorphism (SNP) is strongly associated with cystatin; (B) the SNP is independent of known confounders; and (C) the SNP affects lung cancer exclusively through its impact on cystatin. The study strictly conformed to the proposed STROBE‐MR Statement [17]. The study methodology was provided in Figure 1.

### 2.2 Data Sources

Genetic statistics for NSCLC were sourced from the FINN10 database, which integrates extensive data on genetic variation within the Finnish population. A total of 1627 NSCLC cases and 174 006 health controls were incorporated. Data on squamous cell lung carcinoma and lung adenocarcinoma were derived from an aggregated GWAS study of European descent [18], collected on 7426 cases of squamous cell lung carcinoma and 55 627 controls, as well as 11 273 lung adenocarcinoma cases and 55 483 controls. Genetic data for Cystatin B came from the Milieu Intérieur cohort, a single‐center interventional study of self‐reported Metropolitan French origin spanning three generations [19]. Public GWAS data for Cystatin 8, ‐B, ‐D, ‐F, and ‐M were sourced from the IMPROVE study, a multinational, open‐label, nonrandomized observational study [20]. Detailed SNP information was provided in Table S1, including sample sizes and the number of SNPs. To minimize bias from population stratification, all SNPs and summary data were sourced exclusively from European ancestry populations. No ethical approval was available since publicly accessible data were used in the study.

### 2.3 Selection of IVs

At a threshold of *p* < 5 × 10^−6^, SNPs strongly associated with cystatin were screened due to the lack of SNPs at *p* < 5 × 10^–8^ [21]. SNPs with a minor allele frequency (MAF) ≤ 0.01 were removed from the analysis. Linked disequilibrium (LD)‐clumping was conducted to ensure the independence of each SNP, using stringent criteria (*r* ^2^ < 0.001, window size = 10 000 kb) [22]. For missing SNPs in the outcome summary data, proxy SNPs in high linkage disequilibrium (*R* ^2^ > 0.8) were selected. Palindromic SNPs were removed to address strand orientation and allele coding inconsistencies. Alleles were aligned according to the human genome reference sequence (Build 37) [23]. The strength of each IV was evaluated using the *F*‐statistic, with values > 10 indicating weak instrument bias [24].

### 2.4 MR Analysis

The main method to assess causal relationships between cystatin and lung cancer was the inverse‐variance weighted (IVW) approach. IVW is commonly used in MR analyses to calculate a weighted average effect size, with the inverse variance of each SNP serving as the weight [25]. To ensure validity, additional analyses were performed using MR‐Egger, simple mode, weighted median, and weighted mode approaches. MR‐Egger accounts for intercept terms, offering unbiased causal effect estimates even in the presence of pleiotropy [25]. The simple mode method calculates the causal effect by finding the most frequently occurring value among the estimates derived from each SNP [26]. The weighted median method assumes that half of the IVs are valid, thereby examining causal associations between exposure and outcome [27]. Additionally, the weighted mode method calculates the mode of the effect estimate distribution from individual IVs and assigns corresponding weights [28]. The analyses were conducted using the “TwoSampleMR” package (Version 0.6.23) in R software (Version 4.3.2).

### 2.5 Sensitivity Analysis

Cochran’s Q test assessed heterogeneity based on IVW estimates, with significance defined as *p* < 0.05 [29]. A “leave‐one‐out” sensitivity analysis identified influential SNPs by sequentially excluding each variant [30]. To evaluate horizontal pleiotropy caused by genetic variation, MR‐Egger regression was applied, with pleiotropy absence inferred from near‐zero or statistically insignificant intercepts [31]. The MR pleiotropy residual sum and outlier (MR‐PRESSO) method and the Radial MR method utilizing IVW and MR‐Egger estimates were used to identify outlier SNPs (*p* < 0.05) [32, 33]. Subsequent removal of these outliers allowed for the re‐estimation of causal associations, thus correcting for horizontal pleiotropy. Asymmetry observed in the funnel plot may serve as an indicator of heterogeneity among IVs.

## 3 Results

### 3.1 Selection of IVs

A total of 80 IVs were identified for cystatin, the *F*‐statistics of which all exceeded 10. Eight SNPs absent from the NSCLC summary statistics were replaced with proxy SNPs. Specifically, for lung adenocarcinoma, rs7269564 and rs373361368 were substituted with rs6114913 and rs10404401, respectively. For NSCLC, rs373361368, rs137909368, rs186258664, rs6812091, and rs148479829 were substituted with rs57562054, rs56350477, rs113005217, rs6841027, and rs72928898, respectively. For squamous cell lung carcinoma, rs373361368 was replaced with rs10404401. Detailed information on the IVs was provided in Table S2.

### 3.2 Causal Effects of Cystatin on Lung cancer

**FIGURE 2:**
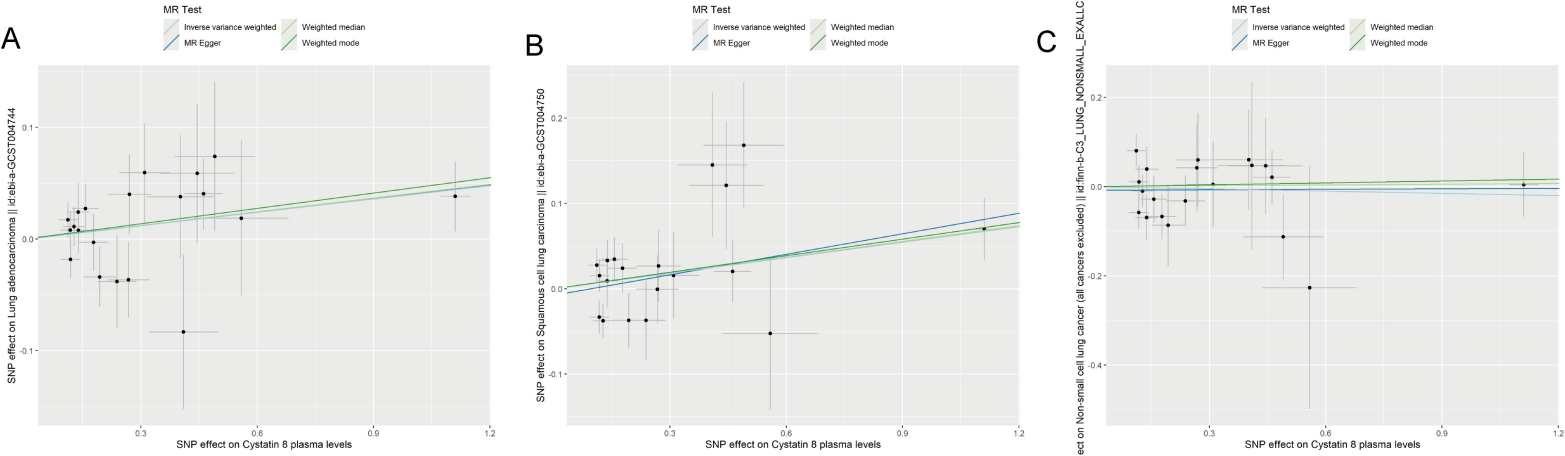
The causal relationships between Cystatin 8 and cell lung cancer using different MR methods. Each panel represents the causal estimates Cystatin 8 for (A) lung adenocarcinoma, (B) squamous cell lung carcinoma, and (C) non‐small cell lung cancer. The slope of each line corresponds to the causal estimates for each method. Individual SNP effects on the outcome (represented by points and vertical lines) against their effects on the exposure (represented by points and horizontal lines) are delineated in the background.

Causal relationship was identified between genetically predicted cystatin 8 and squamous cell lung carcinoma using IVW analysis (OR = 1.062, 95% CI: 1.004–1.124, *p* = 0.035; Table 1). Such causality was validated by weighted median (OR = 1.064, 95% CI: 1.003–1.128, *p* = 0.04) and weighted mode (OR = 1.067, 95% CI: 1.006–1.131, *p* = 0.044) (Table S3). No causal relationships were found for genetically predicted Cystatin B (ebi‐a‐GCST90085722), Cystatin B (prot‐b‐3), Cystatin D, Cystatin F, Cystatin M (prot‐a‐703), or Cystatin M (prot‐a‐704) with squamous cell lung carcinoma (all *p* > 0.05; Table 1). For lung adenocarcinoma and NSCLC, no causal effects were observed for genetically predicted all types of cystatin (all *p* > 0.05). Consistent results were yielded using supplementary methods of causal inference (Table S3). Scatter plots illustrating the effect sizes of SNPs for cystatin on squamous cell lung carcinoma, lung adenocarcinoma, and NSCLC were given in Figure 2. The forest plot also identified causal effects between genetically predicted Cystatin 8 and squamous cell lung carcinoma (Figure S1).

**TABLE 1:** IVW estimates of the causal effects of cystatis on lung cancer.

Exposure	Outcome	Number of SNPs	Methods	OR (95% CI)	P
Cystatin 8	Lung adenocarcinoma	20	Inverse variance weighted	1.041 (0.999–1.084)	0.053
	Non‐small cell lung cancer	20	Inverse variance weighted	0.983 (0.9–1.075)	0.712
	Squamous cell lung carcinoma	19	Inverse variance weighted	1.062 (1.004–1.124)	0.035
Cystatin B (ebi‐a‐GCST90085722)	Lung adenocarcinoma	3	Inverse variance weighted	0.958 (0.815–1.126)	0.605
	Squamous cell lung carcinoma	2	Inverse variance weighted	1.023 (0.621–1.684)	0.93
	Non‐small cell lung cancer	3	Inverse variance weighted	1.121 (0.797–1.578)	0.512
Cystatin B (prot‐b‐3)	Lung adenocarcinoma	3	Inverse variance weighted	0.975 (0.895–1.063)	0.568
	Non‐small cell lung cancer	4	Inverse variance weighted	0.963 (0.864–1.073)	0.494
	Squamous cell lung carcinoma	3	Inverse variance weighted	1.04 (0.956–1.131)	0.366
Cystatin D	Lung adenocarcinoma	10	Inverse variance weighted	1.013 (0.85–1.208)	0.884
	Non‐small cell lung cancer	9	Inverse variance weighted	0.856 (0.651–1.127)	0.269
	Squamous cell lung carcinoma	9	Inverse variance weighted	1.025 (0.916–1.146)	0.67
Cystatin F	Lung adenocarcinoma	13	Inverse variance weighted	1 (0.963–1.038)	0.984
	Non‐small cell lung cancer	13	Inverse variance weighted	1.007 (0.876–1.158)	0.923
	Squamous cell lung carcinoma	13	Inverse variance weighted	1.009 (0.966–1.054)	0.688
Cystatin M (prot‐a‐703)	Lung adenocarcinoma	9	Inverse variance weighted	0.95 (0.865–1.042)	0.274
	Non‐small cell lung cancer	10	Inverse variance weighted	1.082 (0.863–1.357)	0.496
	Squamous cell lung carcinoma	10	Inverse variance weighted	0.999 (0.842–1.184)	0.987
Cystatin M (prot‐a‐704)	Lung adenocarcinoma	13	Inverse variance weighted	0.956 (0.887–1.03)	0.235
	Non‐small cell lung cancer	14	Inverse variance weighted	0.98 (0.833–1.154)	0.813
	Squamous cell lung carcinoma	12	Inverse variance weighted	1.04 (0.936–1.156)	0.463

### 3.3 Sensitivity Analysis

Significant heterogeneity was noted in the relationships between Cystatin D and lung adenocarcinoma (*Q* = 33.837, *p* < 0.001), Cystatin M (prot‐a‐703), and squamous cell lung carcinoma (*Q* = 25.73, *p* = 0.002), and Cystatin F and NSCLC (*Q* = 22.881, *p* = 0.029) (Table 2). Since the IVW method was employed based on random effects, the impacts of heterogeneity can be partially mitigated. Symmetry in the funnel plot suggested no heterogeneity within the causal association between Cystatin 8 and lung cancer (Figure S2). MR‐Egger identified no horizontal pleiotropy in such associations (intercept = −0.055–0.031, all *p* > 0.05) (Table 2). However, horizontal pleiotropy was noted between cystatin D and lung adenocarcinoma (global *p* < 0.001) and between cystatin M (prot‐a‐703) and squamous cell lung carcinoma (global *p* = 0.001) using MR‐PRESSO global test (Table 3). Two SNPs (rs117720979 and rs140668367) and one SNP (rs7908010) were identified as outliers in the above associations. After removing these outliers, a significant causal association was observed between Cystatin D and lung adenocarcinoma (OR = 1.178, 95% CI: 1.023–1.358, *p* = 0.023), while no significant causality was observed between cystatin M (prot‐a‐703) and squamous cell lung carcinoma (OR = 1.069, 95% CI: 0.949–1.206, *p* = 0.272) (Table S4). After outlier removal, significant heterogeneity was resolved for both the association between Cystatin D and lung adenocarcinoma (*Q* = 10.052, *p* = 0.122) and between cystatin M (prot‐a‐703) and squamous cell lung carcinoma (*Q* = 10.134, *p* = 0.256) (Table S5). No pleiotropy was observed for these associations either (MR‐Egger intercept *p* > 0.05 for both; Table S5). In the case of cystatin F and NSCLC, where MR‐PRESSO did not detect any significant outliers, we further investigated potential influential SNPs using Radial MR analysis. This approach identified two outlier SNPs, rs7269564 and rs143377863 (Table S6). Subsequent MR analysis, after excluding these outliers, confirmed the nonsignificant association between cystatin F and NSCLC (OR = 0.940, 95% CI: 0.843–1.048, *p* = 0.264; Table S4). Furthermore, this reanalysis showed no significant heterogeneity (*Q* = 6.868, *p* = 0.738) and no indication of horizontal pleiotropy (Intercept = −0.00509, *p* = 0.878) for this association (Table S5). Moreover, no outlier SNPs were determined in the causal relationship between cystatin 8 and lung cancer using leave‐one‐out analysis (Figure S3). Therefore, sensitivity analyses further validated the robustness of main findings.

**TABLE 2:** Assessing the heterogeneity and horizontal pleiotropy between cystatin and lung cancer.

Exposure	Outcome	Heterogeneity		Horizontal pleiotropy	
		Cochran’s Q	P	MR‐Egger intercept	P
Cystatin B (ebi‐a‐GCST90085722)	Lung adenocarcinoma	1.74	0.419	−0.026	0.61
Cystatin B (ebi‐a‐GCST90085722)	Squamous cell lung carcinoma	3.323	0.068	NA	NA
Cystatin B (ebi‐a‐GCST90085722)	Non‐small cell lung cancer	0.236	0.889	−0.023	0.804
Cystatin D	Lung adenocarcinoma	33.837	< 0.001	0.02	0.597
Cystatin D	Squamous cell lung carcinoma	7.606	0.473	−0.015	0.507
Cystatin D	Non‐small cell lung cancer	14.242	0.076	−0.038	0.527
Cystatin M (prot‐a‐703)	Lung adenocarcinoma	3.466	0.902	−0.012	0.61
Cystatin M (prot‐a‐703)	Squamous cell lung carcinoma	25.73	0.002	0.004	0.924
Cystatin M (prot‐a‐703)	Non‐small cell lung cancer	15.127	0.088	0.031	0.615
Cystatin M (prot‐a‐704)	Lung adenocarcinoma	7.749	0.804	−0.006	0.669
Cystatin M (prot‐a‐704)	Squamous cell lung carcinoma	15.177	0.175	−0.02	0.377
Cystatin M (prot‐a‐704)	Non‐small cell lung cancer	9.762	0.713	−0.015	0.656
Cystatin F	Lung adenocarcinoma	12.733	0.389	−0.012	0.21
Cystatin F	Squamous cell lung carcinoma	12.276	0.424	−0.006	0.604
Cystatin F	Non‐small cell lung cancer	22.881	0.029	−0.005	0.878
Cystatin 8	Lung adenocarcinoma	15.598	0.684	0	0.985
Cystatin 8	Squamous cell lung carcinoma	25.016	0.124	−0.008	0.527
Cystatin 8	Non‐small cell lung cancer	16.343	0.634	−0.008	0.685
Cystatin B (prot‐b‐3)	Lung adenocarcinoma	4.717	0.095	−0.016	0.865
Cystatin B (prot‐b‐3)	Squamous cell lung carcinoma	0.445	0.801	−0.017	0.628
Cystatin B (prot‐b‐3)	Non‐small cell lung cancer	1.821	0.61	−0.055	0.333

*Note:* Cochran’s Q statistic is used for detecting heterogeneity about the IVW estimate.

**TABLE 3:** Detection and correction of horizontal pleiotropy using MR‐PRESSO method.

Exposure	Outcome	Raw		Outlier corrected		Global P	Number of outliers	Distortion P
		OR (CI%)	P	OR (CI%)	P			
Cystatin 8	Lung adenocarcinoma	1.041 (1.003,1.079)	0.046	/	/	0.759	/	0.98
Cystatin 8	Non‐small cell lung cancer	0.983 (0.906,1.068)	0.695	/	/	0.716	/	/
Cystatin 8	Squamous cell lung carcinoma	1.062 (1.004,1.124)	0.05	/	/	0.196	/	/
Cystatin B	Lung adenocarcinoma	0.973 (0.916,1.034)	0.421	/	/	0.377	/	/
Cystatin B	Non‐small cell lung cancer	0.976 (0.91,1.047)	0.528	/	/	0.858	/	/
Cystatin B	Squamous cell lung carcinoma	1.038 (0.96,1.122)	0.401	/	/	0.63	/	/
Cystatin D	Lung adenocarcinoma	1.013 (0.85,1.208)	0.887	1.114 (0.949,1.307)	0.228	< 0.001	2 (rs117720979, rs140668367)	/
Cystatin D	Non‐small cell lung cancer	0.856 (0.651,1.127)	0.301	/	/	0.094	/	/
Cystatin D	Squamous cell lung carcinoma	1.025 (0.919,1.143)	0.674	/	/	0.453	/	/
Cystatin F	Lung adenocarcinoma	1 (0.963,1.038)	0.985	/	/	0.486	/	/
Cystatin F	Non‐small cell lung cancer	1.007 (0.876,1.158)	0.925	/	/	0.058	/	/
Cystatin F	Squamous cell lung carcinoma	1.009 (0.966,1.054)	0.695	/	/	0.509	/	/
Cystatin M (prot‐a‐703)	Lung adenocarcinoma	0.95 (0.893,1.009)	0.135	/	/	0.904	/	/
Cystatin M (prot‐a‐703)	Non‐small cell lung cancer	1.082 (0.863,1.357)	0.513	/	/	0.115	/	/
Cystatin M (prot‐a‐703)	Squamous cell lung carcinoma	0.999 (0.842,1.184)	0.987	1.069 (0.949,1.206)	0.304	0.001	1 (rs7908010)	/
Cystatin M (prot‐a‐704)	Lung adenocarcinoma	0.956 (0.9,1.015)	0.166	/	/	0.781	/	/
Cystatin M (prot‐a‐704)	Non‐small cell lung cancer	0.98 (0.851,1.129)	0.789	/	/	0.678	/	/
Cystatin M (prot‐a‐704)	Squamous cell lung carcinoma	1.04 (0.936,1.156)	0.478	/	/	0.188	/	/

## 4 Discussion

In the two‐sample MR analysis, the IVW analysis revealed positive causal associations between genetically predicted Cystatin 8 and squamous cell lung carcinoma, and cystatin D and lung adenocarcinoma after outlier removal. Despite the observed heterogeneity, SNP outliers, and horizontal pleiotropy, a series of sensitivity analyses confirmed the robustness of the main results.

Cystatin 8 is a member of the cystatin superfamily, which has been implicated in extracellular matrix remodeling and cellular homeostasis [9]. Previous studies have reported higher serum Cystatin 8 concentrations in lung cancer patients compared to healthy individuals [34, 35]. The involvement of Cystatin 8 in protease inhibition can influence cancer cell proliferation and metastasis, providing a plausible mechanistic link to its impact on squamous cell lung carcinoma [36, 37]. Mechanistically, Cystatin 8, secreted by lung cancer cells, could degrade the endothelial glycocalyx, exposing adhesion molecules such as E‐selectin [38]. This enhances cancer cell adhesion to the brain microvasculature, promoting brain metastasis. In this study, our findings revealed a significant causal association between Cystatin 8 and squamous cell lung carcinoma risk via the IVW, weighted median, and weighted mode approaches, suggesting that Cystatin 8 may act as a potential risk factor. Therefore, the role of Cystatin 8 in squamous cell lung carcinoma warrants further investigation to elucidate the exact biological mechanisms. Given its potential as a therapeutic target, Cystatin 8 could pave the way for the development of novel treatments or biomarkers for this cancer subtype. However, further preclinical and clinical studies are needed to validate its therapeutic potential.

The absence of significant causal relationships for other cystatins (B, D, F, M) with squamous cell lung carcinoma, lung adenocarcinoma, and overall NSCLC suggests the isoform‐specific role of cystatins. Moreover, the initial MR analysis revealed heterogeneity for the associations between Cystatin D and lung adenocarcinoma, Cystatin M (prot‐a‐703) and squamous cell lung carcinoma, and Cystatin F and NSCLC. This could be attributed to the outlier SNPs, as the removal of these outliers using MR‐PRESSO and radial analysis removed the heterogeneity. Notably, our findings revealed that Cystatin D plasma levels were causally associated with lung adenocarcinoma risk after outlier removal. This was consistent with previous reports. For example, Cystatin D has been implicated in tumor suppression by inducing mesenchymal–epithelial transition and regulating immune responses [39], even though direct evidence linking Cystatin D and lung cancer was lacking. Nevertheless, it is important to highlight that this positive association was only observed after the exclusion of outliers, so this should be validated in independent cohorts. On the other hand, Cystatins M and F did not exhibit significant association with outcomes even after outlier removal, which contrasted with published population‐based association studies. Emerging evidence indicates that Cystatin B may inhibit tumor invasion through its effects on cathepsin activity and could serve as a prognostic marker in lung cancer [40]. Conversely, Cystatin F is expressed in many cancer cell lines, especially lung carcinoma, and other cells like immune cells [41]. It can be secreted into the tumor microenvironment and inhibit the cytotoxic activity of immune cells, thus contributing to immune evasion [42]. Cystatin M has also demonstrated tumor‐suppressive properties by inhibiting cathepsins and altering gene expression profiles [43]. This highlights the complexity of cystatin functions in cancer biology and underscores the necessity to differentiate between the various isoforms when considering their clinical implications. Moreover, the lack of significant causality may stem from limitations in existing GWAS data, like small sample sizes and weak IVs, which may have obscured potential subtle effects. Future studies should explore the conditions under which these isoforms might trigger cancer progression in the context of the genetic background and interaction with other molecular pathways.

Several strengths of the current study should be acknowledged. First, this research is the first to explore the causality between cystatin and lung cancer. Second, multiple analytical approaches and sensitivity analyses were combined to validate our findings. Third, sufficient statistical power was achieved based on the large sample size, thereby ensuring the reliability of the results. Nonetheless, our study had several limitations. First, the applicability of our results might be limited to populations of European descent, thus extending our findings to other ethnicities should be done with caution. For instance, significant interethnic variations in the allele frequencies of cystatin‐related genes and LD patterns may exist. Moreover, the interaction between environmental exposures, such as smoking and air pollution, and genetic susceptibility can vary across populations. Second, the use of population‐level data limited our ability to stratify populations, potentially masking individual variations. Third, the multifactorial etiology of NSCLC may introduce discrepancies in investigations of cystatin. Finally, the less stringent criteria used for selecting IVs of cystatin may increase the risk of false positives. Refinement of these criteria may enhance the precision of future analyses.

In conclusion, genetically predicted Cystatin 8 was causally associated with squamous cell lung carcinoma. These findings suggest that monitoring Cystatin 8 levels may have potential value in the clinical management of squamous cell lung carcinoma, such as in early detection or disease monitoring. Additionally, further studies are needed to validate its therapeutic potential and to determine the underlying mechanisms contributing to lung cancer.

## Author Contributions

Conception and design: Chunling Zhang and Shihuan Yu. Administrative support: Shihuan Yu. Provision of study materials or patients: Chunling Zhang. Collection and assembly of data: Riya Wu and Hang Liu. Data analysis and interpretation: Chunling Zhang, Riya Wu, and Shihuan Yu. Manuscript writing: all authors. Final approval of manuscript: all authors.

## Ethics Statement

The authors have nothing to report.

## Consent

The authors have nothing to report.

## Conflict of Interest Statement

The authors declare no conflicts of interest.

## Supporting information


**Figure S1** Forrest plot of the causal relationships between Cystatin 8 and cell lung cancer. Each panel represents the causal estimates Cystatin 8 for (A) lung adenocarcinoma, (B) squamous cell lung carcinoma, and (C) non‐small cell lung cancer. The plot visually demonstrates how a single variant influences the causal estimates and the integrated causal estimate of all IVs.
**Figure S2.** The funnel plot of the causal relationships between cystatin 8 and lung cancer. Each panel represents the causal estimates Cystatin 8 for (A) lung adenocarcinoma, (B) squamous cell lung carcinoma, and (C) non‐small cell lung cancer. The plot visually demonstrates the symmetry of effect sizes and their precision to assess publication bias.
**Figure S3.** The leave‐one‐out plot of the causal relationships between cystatin and lung cancer. Each panel represents the Causal Estimates 8 for (A) lung adenocarcinoma, (B) squamous cell lung carcinoma. and (C) non‐small cell lung cancer. The funnel plot illustrated the influence of each IV on the overall meta‐analysis result by recalculating the effect estimate after sequentially omitting each IV.

**Table S1** Overview of the data source.
**Table S2.** Detailed information of IVs in the MR analysis of cystatin on lung cancer.
**Table S3.** MR estimates of assessing the causal effects of cystatin on lung cancer.
**Table S4.** The causal association between cystatin levels and lung cancer after outliers removal.
**Table S5.** The heterogeneity and pleiotropy assessment for the causal association between cystatin levels and lung cancer after outliers removal.
**Table S6.** The identification of outliers in the association between cystatin F levels and NSCLC by Radial MR analysis.

## Data Availability

All data generated or analysed during this study are included in this published article.
