# Supplementary material for: Causal Associations Between Cystatin and Lung Cancer: A Two‐Sample Mendelian Randomization Study
Source: Clin Respir J. 2025 Jul 11;19(7):e70112. doi: 10.1111/crj.70112 (PMC12246730; doi:10.1111/crj.70112)
Supplement: Supplementary file 1 — Figure S1 Forrest plot of the causal relationships between Cystatin 8 and cell lung cancer. Each panel represents the causal estimates Cystatin 8 for (A) lung adenocarcinoma, (B) squamous cell lung carcinoma, and (C) non‐small cell lung cancer. The plot visually demonstrates how a single variant influences the causal estimates and the integrated causal estimate of all IVs. Figure S2. The funnel plot of the causal relationships between cystatin 8 and lung cancer. Each panel represents the causal estimates Cystatin 8 for (A) lung adenocarcinoma, (B) squamous cell lung carcinoma, and (C) non‐small cell lung cancer. The plot visually demonstrates the symmetry of effect sizes and their precision to assess publication bias. Figure S3. The leave‐one‐out plot of the causal relationships between cystatin and lung cancer. Each panel represents the Causal Estimates 8 for (A) lung adenocarcinoma, (B) squamous cell lung carcinoma. and (C) non‐small cell lung cancer. The funnel plot illustrated the influence of each IV on the overall meta‐analysis result by recalculating the effect estimate after sequentially omitting each IV. [file CRJ-19-e70112-s002.docx]

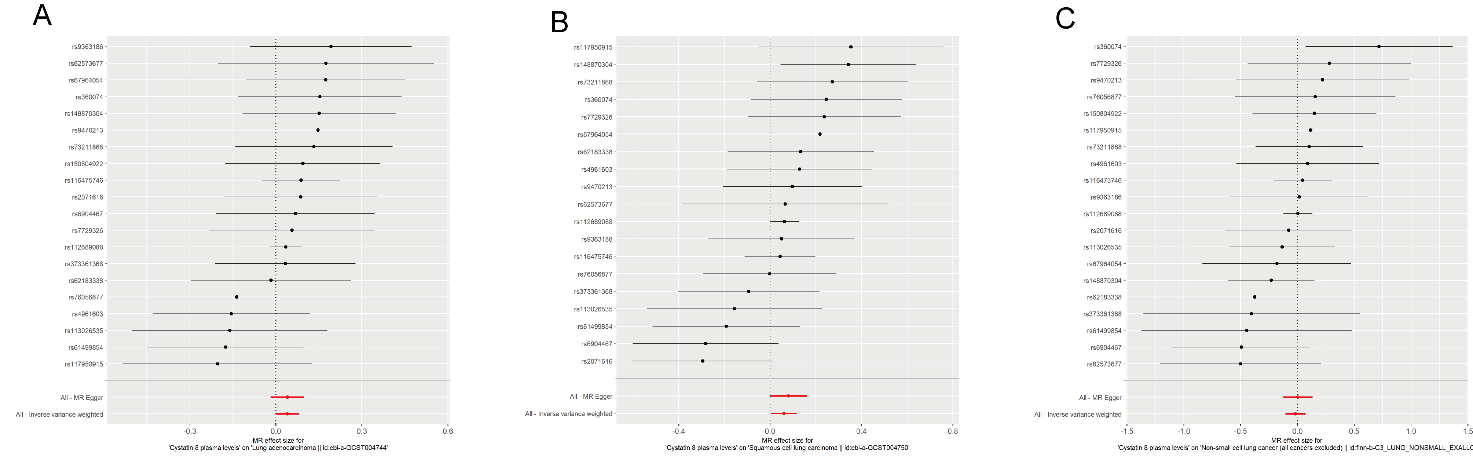
Figure S1. Forrest plot of the causal relationships between Cystatin 8 and cell lung cancer. Each panel represents the causal estimates Cystatin 8 for lung adenocarcinoma (A), squamous cell lung carcinoma (B) and non-small cell lung cancer (C). The plot visually demonstrates how a single variant influences the causal estimates and the integrated causal estimate of all IVs.


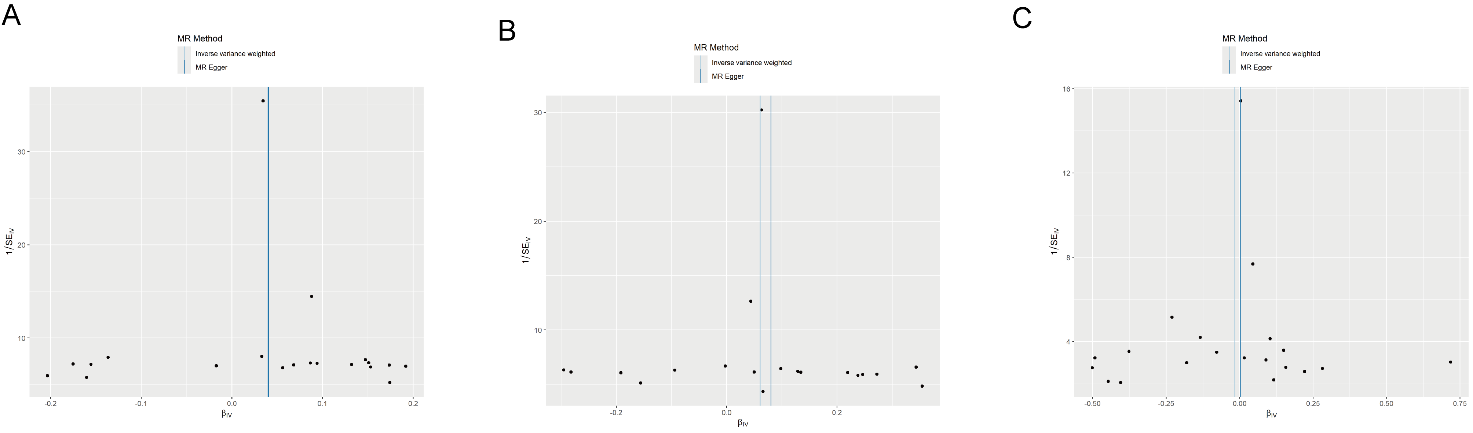


Figure S2. The funnel plot of the causal relationships between cystatin 8 and lung cancer. Each panel represents the causal estimates Cystatin 8 for lung adenocarcinoma (A), squamous cell lung carcinoma (B) and non-small cell lung cancer (C). The plot visually demonstrates the symmetry of effect sizes and their precision to assess publication bias.


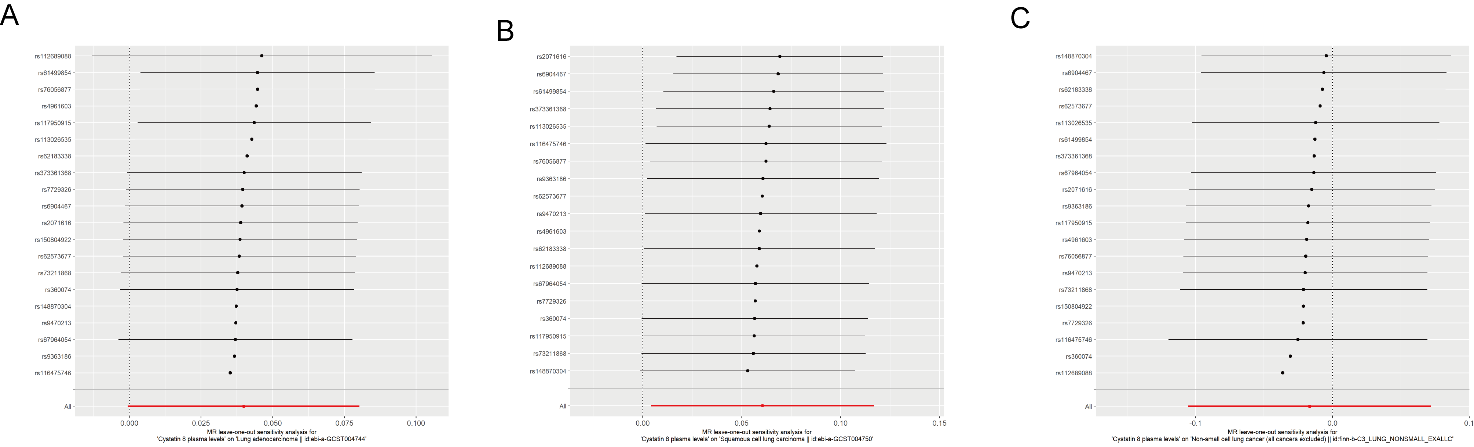


Figure S3. The leave-one-out plot of the causal relationships between cystatin and lung cancer. Each panel represents the causal estimates 8 for lung adenocarcinoma (A), squamous cell lung carcinoma (B) and non-small cell lung cancer (C). The funnel plot illustrated the influence of each IV on the overall meta-analysis result by recalculating the effect estimate after sequentially omitting each IV.
