# Supplementary material for: Causal Associations Between Cystatin and Lung Cancer: A Two‐Sample Mendelian Randomization Study
Source: Clin Respir J. 2025 Jul 11;19(7):e70112. doi: 10.1111/crj.70112 (PMC12246730; doi:10.1111/crj.70112)
Supplement: Supplementary file 2 — Table S1 Overview of the data source. Table S2. Detailed information of IVs in the MR analysis of cystatin on lung cancer. Table S3. MR estimates of assessing the causal effects of cystatin on lung cancer. Table S4. The causal association between cystatin levels and lung cancer after outliers removal. Table S5. The heterogeneity and pleiotropy assessment for the causal association between cystatin levels and lung cancer after outliers removal. Table S6. The identification of outliers in the association between cystatin F levels and NSCLC by Radial MR analysis. [file CRJ-19-e70112-s001.docx]

Table S1. Overview of the data source.

| **Trait** | **GWAS ID** | **Sample size（case/control）** | **Number of SNPs** | **Source** | **Population** |
| --- | --- | --- | --- | --- | --- |
| Non-small cell lung cancer | finn-b-C3_LUNG_NONSMALL_EXALLC | 1,627/174,006 | 16,380,305 | FinnGen | European |
| Squamous cell lung carcinoma | ebi-a-GCST004750 | 7,426/55,627 | 7,838,805 | PMID: 28604730 |  |
| Lung adenocarcinoma | ebi-a-GCST004744 | 11,273/55,483 | 7,849,324 | PMID: 28604730 |  |
| Cystatin B (ebi-a-GCST90085722) | ebi-a-GCST90085722 | 400 | 5,188,525 | PMID: 35264221 |  |
| Cystatin B (prot-b-3) | prot-b-3 | 3,394 | 5,270,646 | the IMPROVE study |  |
| Cystatin-8 | prot-a-706 | 3,301 | 10,534,735 |  |  |
| Cystatin-D | prot-a-702 | 3,301 | 10,534,735 | the IMPROVE study |  |
| Cystatin-F | prot-a-705 | 3,301 | 10,534,735 |  |  |
| Cystatin-M (prot-a-703) | prot-a-703 | 3,301 | 10,534,735 | the IMPROVE study |  |
| Cystatin-M (prot-a-704) | prot-a-704 | 3,301 | 10,534,735 | the IMPROVE study |  |

Table S2. Detailed information of IVs in the MR analysis of cystatin on lung cancer.

| **SNP** | **Chr** | **Pos** | **EA** | **OA** | **EAF** | **Association with cystatin** | | | **Association with outcomes** | | | **r^2^** | **F-statistic** |
| --- | --- | --- | --- | --- | --- | --- | --- | --- | --- | --- | --- | --- | --- |
|  |  |  |  |  |  | **β** | **SE** | **P** | **β** | **SE** | **P** |  |  |
| Instrumental variables of Cystatin 8 on lung adenocarcinoma | | | | | | | | | | | |  |  |
| rs112689088 | 17 | 34307457 | C | T | 0.10 | -1.11 | 0.04 | 6.17E-186 | -0.04 | 0.03 | 0.22 | 0.20 | 844.90 |
| rs113026535 | 3 | 150669396 | G | A | 0.08 | 0.24 | 0.05 | 1.70E-06 | -0.04 | 0.04 | 0.36 | 0.01 | 22.87 |
| rs116475746 | 11 | 71880618 | G | C | 0.07 | 0.46 | 0.05 | 1.35E-21 | 0.04 | 0.03 | 0.20 | 0.03 | 90.90 |
| rs117950915 | 11 | 20814520 | G | A | 0.03 | -0.41 | 0.09 | 3.89E-06 | 0.08 | 0.07 | 0.23 | 0.01 | 21.34 |
| rs148870304 | 14 | 70556525 | A | G | 0.02 | 0.49 | 0.10 | 2.34E-06 | 0.07 | 0.07 | 0.27 | 0.01 | 22.26 |
| rs150804922 | 15 | 51694680 | G | A | 0.02 | 0.40 | 0.09 | 3.63E-06 | 0.04 | 0.06 | 0.49 | 0.01 | 21.44 |
| rs2071616 | 10 | 123279795 | T | C | 0.30 | -0.13 | 0.03 | 3.16E-06 | -0.01 | 0.02 | 0.53 | 0.01 | 21.71 |
| rs360074 | 1 | 226091150 | G | A | 0.59 | -0.11 | 0.02 | 4.37E-06 | -0.02 | 0.02 | 0.29 | 0.01 | 21.09 |
| rs373361368 | 19 | 6887062 | A | G | 0.01 | 0.56 | 0.12 | 4.47E-06 | 0.02 | 0.07 | 0.79 | 0.01 | 21.04 |
| rs4961603 | 9 | 17873229 | G | A | 0.59 | -0.12 | 0.03 | 3.24E-06 | 0.02 | 0.02 | 0.27 | 0.01 | 21.62 |
| rs61499854 | 10 | 29465198 | G | A | 0.10 | -0.19 | 0.04 | 2.82E-06 | 0.03 | 0.03 | 0.21 | 0.01 | 21.97 |
| rs62183338 | 2 | 237969547 | G | C | 0.11 | -0.18 | 0.04 | 2.95E-06 | 0.00 | 0.03 | 0.90 | 0.01 | 21.81 |
| rs62573677 | 9 | 138271005 | T | C | 0.19 | -0.14 | 0.03 | 2.09E-06 | -0.02 | 0.03 | 0.37 | 0.01 | 22.54 |
| rs67964054 | 20 | 8813039 | G | A | 0.16 | -0.16 | 0.03 | 3.72E-06 | -0.03 | 0.02 | 0.22 | 0.01 | 21.39 |
| rs6904467 | 6 | 148489603 | G | A | 0.55 | 0.12 | 0.03 | 2.88E-06 | 0.01 | 0.02 | 0.63 | 0.01 | 21.95 |
| rs73211868 | 12 | 119512172 | A | G | 0.02 | 0.45 | 0.10 | 3.55E-06 | 0.06 | 0.06 | 0.35 | 0.01 | 21.49 |
| rs76056877 | 2 | 71733301 | G | A | 0.08 | -0.27 | 0.05 | 7.94E-07 | 0.04 | 0.03 | 0.28 | 0.01 | 24.36 |
| rs7729326 | 5 | 67506002 | G | A | 0.19 | 0.14 | 0.03 | 2.04E-06 | 0.01 | 0.02 | 0.70 | 0.01 | 22.52 |
| rs9363186 | 6 | 95062937 | A | T | 0.03 | -0.31 | 0.07 | 2.45E-06 | -0.06 | 0.04 | 0.18 | 0.01 | 22.21 |
| rs9470213 | 6 | 11825287 | T | C | 0.05 | -0.27 | 0.06 | 1.62E-06 | -0.04 | 0.04 | 0.26 | 0.01 | 22.94 |
| Instrumental variables of Cystatin 8 on non-small cell lung cancer | | | | | | | | | | | |  |  |
| rs112689088 | 17 | 34307457 | C | T | 0.10 | -1.11 | 0.04 | 6.17E-186 | 0.00 | 0.07 | 0.95 | 0.20 | 844.90 |
| rs113026535 | 3 | 150669396 | G | A | 0.08 | 0.24 | 0.05 | 1.70E-06 | -0.03 | 0.06 | 0.57 | 0.01 | 22.87 |
| rs116475746 | 11 | 71880618 | G | C | 0.07 | 0.46 | 0.05 | 1.35E-21 | 0.02 | 0.06 | 0.73 | 0.03 | 90.90 |
| rs117950915 | 11 | 20814520 | G | A | 0.03 | -0.41 | 0.09 | 3.89E-06 | -0.05 | 0.19 | 0.80 | 0.01 | 21.34 |
| rs148870304 | 14 | 70556525 | A | G | 0.02 | 0.49 | 0.10 | 2.34E-06 | -0.11 | 0.10 | 0.24 | 0.01 | 22.26 |
| rs150804922 | 15 | 51694680 | G | A | 0.02 | 0.40 | 0.09 | 3.63E-06 | 0.06 | 0.11 | 0.59 | 0.01 | 21.44 |
| rs2071616 | 10 | 123279795 | T | C | 0.30 | -0.13 | 0.03 | 3.16E-06 | 0.01 | 0.04 | 0.79 | 0.01 | 21.71 |
| rs360074 | 1 | 226091150 | G | A | 0.59 | -0.11 | 0.02 | 4.37E-06 | -0.08 | 0.04 | 0.03 | 0.01 | 21.09 |
| rs373361368 | 19 | 6889525 | A | G | 0.01 | 0.56 | 0.12 | 4.47E-06 | -0.23 | 0.27 | 0.41 | 0.01 | 21.04 |
| rs4961603 | 9 | 17873229 | G | A | 0.59 | -0.12 | 0.03 | 3.24E-06 | -0.01 | 0.04 | 0.78 | 0.01 | 21.62 |
| rs61499854 | 10 | 29465198 | G | A | 0.10 | -0.19 | 0.04 | 2.82E-06 | 0.09 | 0.09 | 0.34 | 0.01 | 21.97 |
| rs62183338 | 2 | 237969547 | G | C | 0.11 | -0.18 | 0.04 | 2.95E-06 | 0.07 | 0.05 | 0.18 | 0.01 | 21.81 |
| rs62573677 | 9 | 138271005 | T | C | 0.19 | -0.14 | 0.03 | 2.09E-06 | 0.07 | 0.05 | 0.17 | 0.01 | 22.54 |
| rs67964054 | 20 | 8813039 | G | A | 0.16 | -0.16 | 0.03 | 3.72E-06 | 0.03 | 0.05 | 0.59 | 0.01 | 21.39 |
| rs6904467 | 6 | 148489603 | G | A | 0.55 | 0.12 | 0.03 | 2.88E-06 | -0.06 | 0.04 | 0.11 | 0.01 | 21.95 |
| rs73211868 | 12 | 119512172 | A | G | 0.02 | 0.45 | 0.10 | 3.55E-06 | 0.05 | 0.11 | 0.67 | 0.01 | 21.49 |
| rs76056877 | 2 | 71733301 | G | A | 0.08 | -0.27 | 0.05 | 7.94E-07 | -0.04 | 0.10 | 0.66 | 0.01 | 24.36 |
| rs7729326 | 5 | 67506002 | G | A | 0.19 | 0.14 | 0.03 | 2.04E-06 | 0.04 | 0.05 | 0.44 | 0.01 | 22.52 |
| rs9363186 | 6 | 95062937 | A | T | 0.03 | -0.31 | 0.07 | 2.45E-06 | 0.00 | 0.10 | 0.96 | 0.01 | 22.21 |
| rs9470213 | 6 | 11825287 | T | C | 0.05 | -0.27 | 0.06 | 1.62E-06 | -0.06 | 0.11 | 0.57 | 0.01 | 22.94 |
| Instrumental variables of Cystatin 8 on squamous cell lung carcinoma | | | | | | | | | | | |  |  |
| rs112689088 | 17 | 34307457 | C | T | 0.10 | -1.11 | 0.04 | 6.17E-186 | -0.07 | 0.04 | 0.06 | 0.20 | 844.90 |
| rs113026535 | 3 | 150669396 | G | A | 0.08 | 0.24 | 0.05 | 1.70E-06 | -0.04 | 0.05 | 0.42 | 0.01 | 22.87 |
| rs116475746 | 11 | 71880618 | G | C | 0.07 | 0.46 | 0.05 | 1.35E-21 | 0.02 | 0.04 | 0.58 | 0.03 | 90.90 |
| rs117950915 | 11 | 20814520 | G | A | 0.03 | -0.41 | 0.09 | 3.89E-06 | -0.15 | 0.08 | 0.09 | 0.01 | 21.34 |
| rs148870304 | 14 | 70556525 | A | G | 0.02 | 0.49 | 0.10 | 2.34E-06 | 0.17 | 0.07 | 0.02 | 0.01 | 22.26 |
| rs2071616 | 10 | 123279795 | T | C | 0.30 | -0.13 | 0.03 | 3.16E-06 | 0.04 | 0.02 | 0.06 | 0.01 | 21.71 |
| rs360074 | 1 | 226091150 | G | A | 0.59 | -0.11 | 0.02 | 4.37E-06 | -0.03 | 0.02 | 0.14 | 0.01 | 21.09 |
| rs373361368 | 19 | 6887062 | A | G | 0.01 | 0.56 | 0.12 | 4.47E-06 | -0.05 | 0.09 | 0.55 | 0.01 | 21.04 |
| rs4961603 | 9 | 17873229 | G | A | 0.59 | -0.12 | 0.03 | 3.24E-06 | -0.02 | 0.02 | 0.42 | 0.01 | 21.62 |
| rs61499854 | 10 | 29465198 | G | A | 0.10 | -0.19 | 0.04 | 2.82E-06 | 0.04 | 0.03 | 0.24 | 0.01 | 21.97 |
| rs62183338 | 2 | 237969547 | G | C | 0.11 | -0.18 | 0.04 | 2.95E-06 | -0.02 | 0.03 | 0.41 | 0.01 | 21.81 |
| rs62573677 | 9 | 138271005 | T | C | 0.19 | -0.14 | 0.03 | 2.09E-06 | -0.01 | 0.03 | 0.78 | 0.01 | 22.54 |
| rs67964054 | 20 | 8813039 | G | A | 0.16 | -0.16 | 0.03 | 3.72E-06 | -0.03 | 0.03 | 0.18 | 0.01 | 21.39 |
| rs6904467 | 6 | 148489603 | G | A | 0.55 | 0.12 | 0.03 | 2.88E-06 | -0.03 | 0.02 | 0.08 | 0.01 | 21.95 |
| rs73211868 | 12 | 119512172 | A | G | 0.02 | 0.45 | 0.10 | 3.55E-06 | 0.12 | 0.07 | 0.11 | 0.01 | 21.49 |
| rs76056877 | 2 | 71733301 | G | A | 0.08 | -0.27 | 0.05 | 7.94E-07 | 0.00 | 0.04 | 0.99 | 0.01 | 24.36 |
| rs7729326 | 5 | 67506002 | G | A | 0.19 | 0.14 | 0.03 | 2.04E-06 | 0.03 | 0.02 | 0.17 | 0.01 | 22.52 |
| rs9363186 | 6 | 95062937 | A | T | 0.03 | -0.31 | 0.07 | 2.45E-06 | -0.02 | 0.05 | 0.76 | 0.01 | 22.21 |
| rs9470213 | 6 | 11825287 | T | C | 0.05 | -0.27 | 0.06 | 1.62E-06 | -0.03 | 0.04 | 0.53 | 0.01 | 22.94 |
| Instrumental variables of Cystatin B (ebi-a-GCST90085722) on lung adenocarcinoma | | | | | | | | | | | |  |  |
| rs35285321 | 21 | 45201832 | A | G | 0.31 | 0.15 | 0.02 | 2.06E-21 | 0.01 | 0.02 | 0.63 | 0.18 | 89.84 |
| rs3792121 | 2 | 100186996 | C | T | 0.20 | 0.05 | 0.01 | 4.93E-06 | -0.02 | 0.02 | 0.46 | 0.05 | 20.76 |
| rs3819441 | 7 | 81826385 | G | A | 0.31 | 0.14 | 0.03 | 2.63E-06 | -0.02 | 0.02 | 0.27 | 0.05 | 21.96 |
| Instrumental variables of Cystatin B (ebi-a-GCST90085722) on non-small cell lung cancer | | | | | | | | | | | |  |  |
| rs35285321 | 21 | 45201832 | A | G | 0.31 | 0.15 | 0.02 | 2.06E-21 | 0.03 | 0.04 | 0.43 | 0.18 | 89.84 |
| rs3792121 | 2 | 100186996 | C | T | 0.20 | 0.05 | 0.01 | 4.93E-06 | -0.01 | 0.05 | 0.90 | 0.05 | 20.76 |
| rs3819441 | 7 | 81826385 | G | A | 0.31 | 0.14 | 0.03 | 2.63E-06 | 0.01 | 0.04 | 0.86 | 0.05 | 21.96 |
| Instrumental variables of Cystatin B (ebi-a-GCST90085722) on squamous cell lung carcinoma | | | | | | | | | | | |  |  |
| rs3792121 | 2 | 100186996 | C | T | 0.20 | 0.05 | 0.01 | 4.93E-06 | 0.04 | 0.02 | 0.08 | 0.05 | 20.76 |
| rs3819441 | 7 | 81826385 | G | A | 0.31 | 0.14 | 0.03 | 2.63E-06 | -0.01 | 0.02 | 0.67 | 0.05 | 21.96 |
| Instrumental variables of Cystatin B (prot-b-3) on lung adenocarcinoma | | | | | | | | | | | |  |  |
| rs149081536 | 7 | 70811324 | G | A | 0.02 | 1.70 | 0.34 | 8.10E-07 | 0.02 | 0.08 | 0.79 | 0.01 | 24.41 |
| rs35285321 | 21 | 45201832 | A | G | 0.31 | -0.43 | 0.03 | 1.16E-43 | 0.01 | 0.02 | 0.63 | 0.06 | 197.55 |
| rs72643661 | 4 | 68701420 | A | T | 0.03 | 0.67 | 0.15 | 4.04E-06 | -0.14 | 0.06 | 0.02 | 0.01 | 21.29 |
| Instrumental variables of Cystatin B (prot-b-3) on non-small cell lung cancer | | | | | | | | | | | |  |  |
| rs149081536 | 7 | 70811324 | G | A | 0.02 | 1.70 | 0.34 | 8.10E-07 | 0.01 | 0.15 | 0.92 | 0.01 | 24.41 |
| rs28615487 | 4 | 104360812 | T | A | 0.30 | 0.18 | 0.04 | 3.99E-06 | -0.05 | 0.04 | 0.21 | 0.01 | 21.33 |
| rs35285321 | 21 | 45201832 | A | G | 0.31 | -0.43 | 0.03 | 1.16E-43 | 0.03 | 0.04 | 0.43 | 0.06 | 197.55 |
| rs72643661 | 4 | 68701420 | A | T | 0.03 | 0.67 | 0.15 | 4.04E-06 | 0.03 | 0.10 | 0.80 | 0.01 | 21.29 |
| Instrumental variables of Cystatin B (prot-b-3) on squamous cell lung carcinoma | | | | | | | | | | | |  |  |
| rs149081536 | 7 | 70811324 | G | A | 0.02 | 1.70 | 0.34 | 8.10E-07 | 0.09 | 0.09 | 0.29 | 0.01 | 24.41 |
| rs28615487 | 4 | 104360812 | T | A | 0.30 | 0.18 | 0.04 | 3.99E-06 | -0.01 | 0.02 | 0.80 | 0.01 | 21.33 |
| rs72643661 | 4 | 68701420 | A | T | 0.03 | 0.67 | 0.15 | 4.04E-06 | 0.02 | 0.07 | 0.78 | 0.01 | 21.29 |
| Instrumental variables of Cystatin D on lung adenocarcinoma | | | | | | | | | | | |  |  |
| rs117720979 | 8 | 123234714 | T | C | 0.01 | 0.46 | 0.09 | 1.07E-06 | -0.17 | 0.07 | 0.01 | 0.01 | 23.80 |
| rs11902678 | 2 | 207815104 | G | A | 0.45 | 0.12 | 0.02 | 3.47E-06 | 0.02 | 0.02 | 0.14 | 0.01 | 21.56 |
| rs13425181 | 2 | 233809622 | A | G | 0.08 | -0.22 | 0.05 | 4.68E-06 | -0.08 | 0.03 | 0.02 | 0.01 | 20.92 |
| rs137909368 | 4 | 3478019 | C | T | 0.13 | 0.16 | 0.03 | 2.45E-06 | -0.02 | 0.02 | 0.40 | 0.01 | 22.23 |
| rs138044143 | 8 | 21170728 | G | C | 0.02 | 0.37 | 0.08 | 8.71E-07 | 0.19 | 0.09 | 0.03 | 0.01 | 24.18 |
| rs140668367 | 2 | 27569612 | C | A | 0.03 | -0.41 | 0.08 | 6.17E-07 | 0.14 | 0.06 | 0.02 | 0.01 | 24.88 |
| rs148393709 | 1 | 202070898 | T | C | 0.01 | 0.87 | 0.18 | 2.29E-06 | 0.10 | 0.11 | 0.36 | 0.01 | 22.35 |
| rs62020722 | 15 | 101263595 | C | T | 0.46 | 0.12 | 0.03 | 4.27E-06 | 0.00 | 0.02 | 0.99 | 0.01 | 21.21 |
| rs76017623 | 2 | 47571063 | T | C | 0.09 | -0.21 | 0.04 | 1.07E-06 | 0.05 | 0.03 | 0.08 | 0.01 | 23.77 |
| rs9788072 | 12 | 4905214 | G | A | 0.09 | 0.23 | 0.05 | 1.41E-06 | 0.06 | 0.03 | 0.04 | 0.01 | 23.29 |
| Instrumental variables of Cystatin D on non-small cell lung cancer | | | | | | | | | | | |  |  |
| rs117720979 | 8 | 123234714 | T | C | 0.01 | 0.46 | 0.09 | 1.07E-06 | 0.02 | 0.22 | 0.94 | 0.01 | 23.80 |
| rs11902678 | 2 | 207815104 | G | A | 0.45 | 0.12 | 0.02 | 3.47E-06 | 0.04 | 0.04 | 0.29 | 0.01 | 21.56 |
| rs13425181 | 2 | 233809622 | A | G | 0.08 | -0.22 | 0.05 | 4.68E-06 | -0.05 | 0.07 | 0.50 | 0.01 | 20.92 |
| rs137909368 | 4 | 3482342 | C | T | 0.13 | 0.16 | 0.03 | 2.45E-06 | 0.01 | 0.06 | 0.92 | 0.01 | 22.23 |
| rs140668367 | 2 | 27569612 | C | A | 0.03 | -0.41 | 0.08 | 6.17E-07 | 0.14 | 0.10 | 0.17 | 0.01 | 24.88 |
| rs148393709 | 1 | 202070898 | T | C | 0.01 | 0.87 | 0.18 | 2.29E-06 | 0.19 | 0.27 | 0.48 | 0.01 | 22.35 |
| rs62020722 | 15 | 101263595 | C | T | 0.46 | 0.12 | 0.03 | 4.27E-06 | -0.12 | 0.04 | 0.00 | 0.01 | 21.21 |
| rs76017623 | 2 | 47571063 | T | C | 0.09 | -0.21 | 0.04 | 1.07E-06 | 0.08 | 0.06 | 0.17 | 0.01 | 23.77 |
| rs9788072 | 12 | 4905214 | G | A | 0.09 | 0.23 | 0.05 | 1.41E-06 | -0.04 | 0.08 | 0.65 | 0.01 | 23.29 |
| Instrumental variables of Cystatin D on squamous cell lung carcinoma | | | | | | | | | | | |  |  |
| rs117720979 | 8 | 123234714 | T | C | 0.01 | 0.46 | 0.09 | 1.07E-06 | 0.02 | 0.08 | 0.82 | 0.01 | 23.80 |
| rs11902678 | 2 | 207815104 | G | A | 0.45 | 0.12 | 0.02 | 3.47E-06 | 0.03 | 0.02 | 0.14 | 0.01 | 21.56 |
| rs13425181 | 2 | 233809622 | A | G | 0.08 | -0.22 | 0.05 | 4.68E-06 | 0.02 | 0.04 | 0.67 | 0.01 | 20.92 |
| rs137909368 | 4 | 3478019 | C | T | 0.13 | 0.16 | 0.03 | 2.45E-06 | -0.03 | 0.03 | 0.31 | 0.01 | 22.23 |
| rs138044143 | 8 | 21170728 | G | C | 0.02 | 0.37 | 0.08 | 8.71E-07 | 0.07 | 0.11 | 0.55 | 0.01 | 24.18 |
| rs140668367 | 2 | 27569612 | C | A | 0.03 | -0.41 | 0.08 | 6.17E-07 | -0.05 | 0.07 | 0.48 | 0.01 | 24.88 |
| rs148393709 | 1 | 202070898 | T | C | 0.01 | 0.87 | 0.18 | 2.29E-06 | 0.05 | 0.13 | 0.70 | 0.01 | 22.35 |
| rs62020722 | 15 | 101263595 | C | T | 0.46 | 0.12 | 0.03 | 4.27E-06 | -0.03 | 0.02 | 0.11 | 0.01 | 21.21 |
| rs76017623 | 2 | 47571063 | T | C | 0.09 | -0.21 | 0.04 | 1.07E-06 | -0.03 | 0.03 | 0.37 | 0.01 | 23.77 |
| Instrumental variables of Cystatin F on lung adenocarcinoma | | | | | | | | | | | |  |  |
| rs113656409 | 20 | 25003294 | G | A | 0.03 | 1.39 | 0.08 | 1.55E-69 | 0.02 | 0.06 | 0.68 | 0.09 | 310.76 |
| rs143377863 | 19 | 49768064 | T | A | 0.02 | 0.37 | 0.07 | 7.59E-07 | -0.07 | 0.06 | 0.27 | 0.01 | 24.49 |
| rs1706477 | 12 | 123913697 | G | T | 0.44 | 0.15 | 0.03 | 1.41E-06 | -0.04 | 0.02 | 0.04 | 0.01 | 23.25 |
| rs186123558 | 17 | 12997388 | T | C | 0.01 | -0.56 | 0.12 | 1.74E-06 | 0.02 | 0.08 | 0.78 | 0.01 | 22.87 |
| rs186258664 | 19 | 21844201 | T | C | 0.06 | -0.23 | 0.05 | 3.09E-06 | -0.04 | 0.04 | 0.21 | 0.01 | 21.75 |
| rs2387362 | 20 | 24899906 | T | C | 0.79 | 0.76 | 0.03 | 1.07E-189 | 0.01 | 0.02 | 0.59 | 0.21 | 865.39 |
| rs4894484 | 3 | 174986124 | A | G | 0.30 | -0.14 | 0.03 | 3.63E-06 | 0.00 | 0.02 | 0.89 | 0.01 | 21.41 |
| rs57832775 | 9 | 109075787 | T | C | 0.09 | -0.22 | 0.05 | 4.90E-06 | 0.04 | 0.03 | 0.19 | 0.01 | 20.86 |
| rs6577309 | 1 | 102714517 | C | T | 0.67 | 0.13 | 0.03 | 1.48E-06 | 0.00 | 0.02 | 0.99 | 0.01 | 23.20 |
| rs6812091 | 4 | 173079572 | G | A | 0.39 | -0.12 | 0.03 | 3.63E-06 | 0.01 | 0.02 | 0.54 | 0.01 | 21.44 |
| rs6993770 | 8 | 106581528 | T | A | 0.30 | -0.17 | 0.03 | 6.17E-10 | 0.03 | 0.02 | 0.13 | 0.01 | 38.20 |
| rs7269564 | 20 | 24882966 | C | T | 0.36 | 0.25 | 0.03 | 1.02E-22 | 0.01 | 0.02 | 0.63 | 0.03 | 95.86 |
| rs7297614 | 12 | 107497313 | T | C | 0.63 | 0.13 | 0.03 | 3.63E-07 | 0.01 | 0.02 | 0.46 | 0.01 | 25.89 |
| Instrumental variables of Cystatin F on non-small cell lung cancer | | | | | | | | | | | |  |  |
| rs111825697 | 22 | 37749536 | A | G | 0.01 | -0.58 | 0.12 | 2.04E-06 | 0.45 | 0.41 | 0.27 | 0.01 | 22.55 |
| rs113656409 | 20 | 25003294 | G | A | 0.03 | 1.39 | 0.08 | 1.55E-69 | 0.19 | 0.24 | 0.43 | 0.09 | 310.76 |
| rs143377863 | 19 | 49768064 | T | A | 0.02 | 0.37 | 0.07 | 7.59E-07 | 0.41 | 0.12 | 0.00 | 0.01 | 24.49 |
| rs183113549 | 20 | 47950861 | G | A | 0.01 | 0.56 | 0.11 | 3.98E-07 | -0.05 | 0.11 | 0.62 | 0.01 | 25.69 |
| rs186258664 | 19 | 21845172 | T | C | 0.06 | -0.23 | 0.05 | 3.09E-06 | 0.06 | 0.06 | 0.26 | 0.01 | 21.75 |
| rs2387362 | 20 | 24899906 | T | C | 0.79 | 0.76 | 0.03 | 1.07E-189 | -0.04 | 0.06 | 0.48 | 0.21 | 865.39 |
| rs4894484 | 3 | 174986124 | A | G | 0.30 | -0.14 | 0.03 | 3.63E-06 | 0.04 | 0.04 | 0.32 | 0.01 | 21.41 |
| rs57832775 | 9 | 109075787 | T | C | 0.09 | -0.22 | 0.05 | 4.90E-06 | 0.00 | 0.06 | 0.98 | 0.01 | 20.86 |
| rs6577309 | 1 | 102714517 | C | T | 0.67 | 0.13 | 0.03 | 1.48E-06 | -0.03 | 0.04 | 0.48 | 0.01 | 23.20 |
| rs6812091 | 4 | 173080590 | G | A | 0.39 | -0.12 | 0.03 | 3.63E-06 | 0.02 | 0.04 | 0.56 | 0.01 | 21.44 |
| rs6993770 | 8 | 106581528 | T | A | 0.30 | -0.17 | 0.03 | 6.17E-10 | 0.04 | 0.04 | 0.38 | 0.01 | 38.20 |
| rs7269564 | 20 | 24881393 | C | T | 0.36 | 0.25 | 0.03 | 1.02E-22 | 0.08 | 0.04 | 0.04 | 0.03 | 95.86 |
| rs7297614 | 12 | 107497313 | T | C | 0.63 | 0.13 | 0.03 | 3.63E-07 | 0.05 | 0.04 | 0.19 | 0.01 | 25.89 |
| Instrumental variables of Cystatin F on squamous cell lung carcinoma | | | | | | | | | | | |  |  |
| rs113656409 | 20 | 25003294 | G | A | 0.03 | 1.39 | 0.08 | 1.55E-69 | 0.04 | 0.07 | 0.58 | 0.09 | 310.76 |
| rs143377863 | 19 | 49768064 | T | A | 0.02 | 0.37 | 0.07 | 7.59E-07 | 0.09 | 0.07 | 0.19 | 0.01 | 24.49 |
| rs1706477 | 12 | 123913697 | G | T | 0.44 | 0.15 | 0.03 | 1.41E-06 | 0.03 | 0.02 | 0.13 | 0.01 | 23.25 |
| rs186123558 | 17 | 12997388 | T | C | 0.01 | -0.56 | 0.12 | 1.74E-06 | 0.03 | 0.09 | 0.73 | 0.01 | 22.87 |
| rs186258664 | 19 | 21844201 | T | C | 0.06 | -0.23 | 0.05 | 3.09E-06 | 0.02 | 0.04 | 0.62 | 0.01 | 21.75 |
| rs2387362 | 20 | 24899906 | T | C | 0.79 | 0.76 | 0.03 | 1.07E-189 | 0.02 | 0.02 | 0.50 | 0.21 | 865.39 |
| rs4894484 | 3 | 174986124 | A | G | 0.30 | -0.14 | 0.03 | 3.63E-06 | -0.01 | 0.02 | 0.64 | 0.01 | 21.41 |
| rs57832775 | 9 | 109075787 | T | C | 0.09 | -0.22 | 0.05 | 4.90E-06 | 0.02 | 0.04 | 0.62 | 0.01 | 20.86 |
| rs6577309 | 1 | 102714517 | C | T | 0.67 | 0.13 | 0.03 | 1.48E-06 | -0.02 | 0.02 | 0.23 | 0.01 | 23.20 |
| rs6812091 | 4 | 173079572 | G | A | 0.39 | -0.12 | 0.03 | 3.63E-06 | -0.02 | 0.02 | 0.28 | 0.01 | 21.44 |
| rs6993770 | 8 | 106581528 | T | A | 0.30 | -0.17 | 0.03 | 6.17E-10 | 0.02 | 0.02 | 0.42 | 0.01 | 38.20 |
| rs7269564 | 20 | 24881393 | C | T | 0.36 | 0.25 | 0.03 | 1.02E-22 | -0.05 | 0.03 | 0.07 | 0.03 | 95.86 |
| rs7297614 | 12 | 107497313 | T | C | 0.63 | 0.13 | 0.03 | 3.63E-07 | -0.01 | 0.02 | 0.65 | 0.01 | 25.89 |
| Instrumental variables of Cystatin M (prot-a-703) on lung adenocarcinoma | | | | | | | | | | | |  |  |
| rs13056506 | 22 | 38580917 | T | G | 0.59 | 0.11 | 0.02 | 4.27E-06 | -0.01 | 0.02 | 0.54 | 0.01 | 21.08 |
| rs144973816 | 12 | 40290267 | C | T | 0.01 | 0.42 | 0.09 | 2.57E-06 | -0.09 | 0.07 | 0.19 | 0.01 | 22.10 |
| rs145533598 | 3 | 12373486 | A | G | 0.01 | 0.46 | 0.10 | 1.55E-06 | -0.03 | 0.07 | 0.67 | 0.01 | 23.06 |
| rs17601330 | 1 | 187410465 | T | C | 0.02 | -0.32 | 0.07 | 3.63E-06 | -0.02 | 0.05 | 0.67 | 0.01 | 21.42 |
| rs35167224 | 18 | 31610104 | C | A | 0.02 | -0.46 | 0.09 | 6.17E-07 | -0.06 | 0.08 | 0.45 | 0.01 | 24.86 |
| rs3825068 | 11 | 65768093 | G | A | 0.03 | 0.57 | 0.08 | 3.98E-14 | -0.02 | 0.05 | 0.74 | 0.02 | 57.14 |
| rs72858535 | 1 | 10724421 | T | C | 0.02 | -0.36 | 0.08 | 1.48E-06 | 0.03 | 0.06 | 0.56 | 0.01 | 23.15 |
| rs7908010 | 10 | 4955910 | G | C | 0.89 | 0.19 | 0.04 | 3.24E-06 | -0.03 | 0.03 | 0.27 | 0.01 | 21.66 |
| rs80293268 | 1 | 8207579 | C | G | 0.05 | -0.30 | 0.06 | 2.14E-07 | 0.00 | 0.05 | 0.96 | 0.01 | 26.88 |
| Instrumental variables of Cystatin M (prot-a-703) on non-small cell lung cancer | | | | | | | | | | | |  |  |
| rs13056506 | 22 | 38580917 | T | G | 0.59 | 0.11 | 0.02 | 4.27E-06 | -0.01 | 0.04 | 0.72 | 0.01 | 21.08 |
| rs145533598 | 3 | 12373486 | A | G | 0.01 | 0.46 | 0.10 | 1.55E-06 | -0.04 | 0.13 | 0.75 | 0.01 | 23.06 |
| rs17601330 | 1 | 187410465 | T | C | 0.02 | -0.32 | 0.07 | 3.63E-06 | -0.14 | 0.10 | 0.15 | 0.01 | 21.42 |
| rs35167224 | 18 | 31610104 | C | A | 0.02 | -0.46 | 0.09 | 6.17E-07 | 0.47 | 0.22 | 0.03 | 0.01 | 24.86 |
| rs3825068 | 11 | 65768093 | G | A | 0.03 | 0.57 | 0.08 | 3.98E-14 | 0.13 | 0.12 | 0.28 | 0.02 | 57.14 |
| rs72858535 | 1 | 10724421 | T | C | 0.02 | -0.36 | 0.08 | 1.48E-06 | 0.24 | 0.23 | 0.30 | 0.01 | 23.15 |
| rs73034086 | 19 | 36548964 | G | T | 0.01 | 0.65 | 0.14 | 2.24E-06 | -0.11 | 0.15 | 0.43 | 0.01 | 22.35 |
| rs73168309 | 12 | 102200018 | G | T | 0.07 | 0.22 | 0.05 | 1.20E-06 | -0.04 | 0.08 | 0.65 | 0.01 | 23.56 |
| rs7908010 | 10 | 4955910 | G | C | 0.89 | 0.19 | 0.04 | 3.24E-06 | 0.11 | 0.05 | 0.02 | 0.01 | 21.66 |
| rs80293268 | 1 | 8207579 | C | G | 0.05 | -0.30 | 0.06 | 2.14E-07 | -0.06 | 0.07 | 0.39 | 0.01 | 26.88 |
| Instrumental variables of Cystatin M (prot-a-703) on squamous cell lung carcinoma | | | | | | | | | | | |  |  |
| rs13056506 | 22 | 38580917 | T | G | 0.59 | 0.11 | 0.02 | 4.27E-06 | 0.04 | 0.02 | 0.03 | 0.01 | 21.08 |
| rs144973816 | 12 | 40290267 | C | T | 0.01 | 0.42 | 0.09 | 2.57E-06 | 0.03 | 0.07 | 0.64 | 0.01 | 22.10 |
| rs145533598 | 3 | 12373486 | A | G | 0.01 | 0.46 | 0.10 | 1.55E-06 | 0.12 | 0.08 | 0.14 | 0.01 | 23.06 |
| rs17601330 | 1 | 187410465 | T | C | 0.02 | -0.32 | 0.07 | 3.63E-06 | -0.08 | 0.06 | 0.18 | 0.01 | 21.42 |
| rs35167224 | 18 | 31610104 | C | A | 0.02 | -0.46 | 0.09 | 6.17E-07 | 0.09 | 0.10 | 0.37 | 0.01 | 24.86 |
| rs3825068 | 11 | 65768093 | G | A | 0.03 | 0.57 | 0.08 | 3.98E-14 | 0.03 | 0.06 | 0.56 | 0.02 | 57.14 |
| rs72858535 | 1 | 10724421 | T | C | 0.02 | -0.36 | 0.08 | 1.48E-06 | 0.08 | 0.07 | 0.26 | 0.01 | 23.15 |
| rs73168309 | 12 | 102200018 | G | T | 0.07 | 0.22 | 0.05 | 1.20E-06 | -0.02 | 0.04 | 0.64 | 0.01 | 23.56 |
| rs7908010 | 10 | 4955910 | G | C | 0.89 | 0.19 | 0.04 | 3.24E-06 | -0.11 | 0.03 | 0.00 | 0.01 | 21.66 |
| rs80293268 | 1 | 8207579 | C | G | 0.05 | -0.30 | 0.06 | 2.14E-07 | 0.01 | 0.06 | 0.88 | 0.01 | 26.88 |
| Instrumental variables of Cystatin M (prot-a-704) on lung adenocarcinoma | | | | | | | | | | | |  |  |
| rs12675687 | 8 | 142253369 | T | C | 0.18 | -0.15 | 0.03 | 4.90E-06 | 0.04 | 0.02 | 0.05 | 0.01 | 20.90 |
| rs144973816 | 12 | 40290267 | C | T | 0.01 | 0.41 | 0.09 | 4.17E-06 | -0.09 | 0.07 | 0.19 | 0.01 | 21.18 |
| rs148479829 | 11 | 65753833 | T | C | 0.03 | 0.59 | 0.08 | 3.16E-15 | 0.01 | 0.05 | 0.84 | 0.02 | 62.05 |
| rs17072386 | 5 | 169696022 | A | G | 0.04 | -0.25 | 0.05 | 3.02E-06 | -0.02 | 0.05 | 0.74 | 0.01 | 21.83 |
| rs2984440 | 6 | 39894628 | G | A | 0.45 | -0.12 | 0.03 | 2.63E-06 | 0.01 | 0.02 | 0.49 | 0.01 | 22.10 |
| rs4690150 | 4 | 81211570 | C | G | 0.71 | 0.13 | 0.03 | 4.17E-06 | -0.02 | 0.02 | 0.34 | 0.01 | 21.20 |
| rs73696763 | 8 | 101451910 | A | T | 0.10 | -0.22 | 0.05 | 3.24E-06 | 0.03 | 0.04 | 0.42 | 0.01 | 21.67 |
| rs77086255 | 18 | 35237318 | C | G | 0.02 | -0.47 | 0.10 | 1.70E-06 | 0.05 | 0.07 | 0.51 | 0.01 | 22.91 |
| rs7805319 | 7 | 148696169 | T | C | 0.21 | 0.15 | 0.03 | 4.37E-06 | 0.00 | 0.02 | 0.81 | 0.01 | 21.10 |
| rs78447376 | 3 | 179843466 | C | A | 0.06 | 0.30 | 0.06 | 6.76E-07 | 0.02 | 0.04 | 0.56 | 0.01 | 24.62 |
| rs78924983 | 1 | 8204596 | T | C | 0.05 | -0.33 | 0.06 | 5.50E-09 | 0.01 | 0.05 | 0.85 | 0.01 | 33.97 |
| rs9602139 | 13 | 83839263 | T | C | 0.31 | 0.12 | 0.03 | 4.57E-06 | -0.01 | 0.02 | 0.70 | 0.01 | 21.09 |
| rs9895630 | 17 | 15012905 | A | G | 0.51 | 0.12 | 0.03 | 2.19E-06 | 0.01 | 0.02 | 0.50 | 0.01 | 22.45 |
| Instrumental variables of Cystatin M (prot-a-704) on non-small cell lung cancer | | | | | | | | | | | |  |  |
| rs12675687 | 8 | 142253369 | T | C | 0.18 | -0.15 | 0.03 | 4.90E-06 | 0.04 | 0.05 | 0.43 | 0.01 | 20.90 |
| rs139642297 | 16 | 61359573 | A | G | 0.01 | 0.56 | 0.12 | 3.47E-06 | 0.06 | 0.30 | 0.83 | 0.01 | 21.55 |
| rs148479829 | 11 | 65757290 | T | C | 0.03 | 0.59 | 0.08 | 3.16E-15 | 0.13 | 0.12 | 0.27 | 0.02 | 62.05 |
| rs17072386 | 5 | 169696022 | A | G | 0.04 | -0.25 | 0.05 | 3.02E-06 | 0.02 | 0.11 | 0.84 | 0.01 | 21.83 |
| rs2984440 | 6 | 39894628 | G | A | 0.45 | -0.12 | 0.03 | 2.63E-06 | 0.00 | 0.04 | 0.99 | 0.01 | 22.10 |
| rs4690150 | 4 | 81211570 | C | G | 0.71 | 0.13 | 0.03 | 4.17E-06 | 0.04 | 0.04 | 0.33 | 0.01 | 21.20 |
| rs72919535 | 1 | 37418571 | C | T | 0.01 | 0.75 | 0.16 | 2.51E-06 | -0.44 | 0.33 | 0.18 | 0.01 | 22.15 |
| rs73696763 | 8 | 101451910 | A | T | 0.10 | -0.22 | 0.05 | 3.24E-06 | 0.06 | 0.06 | 0.29 | 0.01 | 21.67 |
| rs77086255 | 18 | 35237318 | C | G | 0.02 | -0.47 | 0.10 | 1.70E-06 | 0.31 | 0.32 | 0.32 | 0.01 | 22.91 |
| rs7805319 | 7 | 148696169 | T | C | 0.21 | 0.15 | 0.03 | 4.37E-06 | -0.07 | 0.05 | 0.15 | 0.01 | 21.10 |
| rs78447376 | 3 | 179843466 | C | A | 0.06 | 0.30 | 0.06 | 6.76E-07 | -0.02 | 0.11 | 0.86 | 0.01 | 24.62 |
| rs78924983 | 1 | 8204596 | T | C | 0.05 | -0.33 | 0.06 | 5.50E-09 | -0.06 | 0.07 | 0.36 | 0.01 | 33.97 |
| rs9602139 | 13 | 83839263 | T | C | 0.31 | 0.12 | 0.03 | 4.57E-06 | 0.01 | 0.04 | 0.87 | 0.01 | 21.09 |
| rs9895630 | 17 | 15012905 | A | G | 0.51 | 0.12 | 0.03 | 2.19E-06 | -0.01 | 0.04 | 0.85 | 0.01 | 22.45 |
| Instrumental variables of Cystatin M (prot-a-704) on squamous cell lung carcinoma | | | | | | | | | | | |  |  |
| rs12675687 | 8 | 142253369 | T | C | 0.18 | -0.15 | 0.03 | 4.90E-06 | 0.02 | 0.02 | 0.48 | 0.01 | 20.90 |
| rs144973816 | 12 | 40290267 | C | T | 0.01 | 0.41 | 0.09 | 4.17E-06 | 0.03 | 0.07 | 0.64 | 0.01 | 21.18 |
| rs148479829 | 11 | 65753833 | T | C | 0.03 | 0.59 | 0.08 | 3.16E-15 | 0.03 | 0.06 | 0.54 | 0.02 | 62.05 |
| rs17072386 | 5 | 169696022 | A | G | 0.04 | -0.25 | 0.05 | 3.02E-06 | 0.07 | 0.06 | 0.27 | 0.01 | 21.83 |
| rs2984440 | 6 | 39894628 | G | A | 0.45 | -0.12 | 0.03 | 2.63E-06 | 0.02 | 0.02 | 0.38 | 0.01 | 22.10 |
| rs4690150 | 4 | 81211570 | C | G | 0.71 | 0.13 | 0.03 | 4.17E-06 | 0.05 | 0.02 | 0.02 | 0.01 | 21.20 |
| rs73696763 | 8 | 101451910 | A | T | 0.10 | -0.22 | 0.05 | 3.24E-06 | -0.02 | 0.04 | 0.73 | 0.01 | 21.67 |
| rs77086255 | 18 | 35237318 | C | G | 0.02 | -0.47 | 0.10 | 1.70E-06 | -0.06 | 0.09 | 0.51 | 0.01 | 22.91 |
| rs7805319 | 7 | 148696169 | T | C | 0.21 | 0.15 | 0.03 | 4.37E-06 | 0.01 | 0.02 | 0.78 | 0.01 | 21.10 |
| rs78447376 | 3 | 179843466 | C | A | 0.06 | 0.30 | 0.06 | 6.76E-07 | 0.10 | 0.05 | 0.04 | 0.01 | 24.62 |
| rs78924983 | 1 | 8204596 | T | C | 0.05 | -0.33 | 0.06 | 5.50E-09 | 0.01 | 0.06 | 0.82 | 0.01 | 33.97 |
| rs9602139 | 13 | 83839263 | T | C | 0.31 | 0.12 | 0.03 | 4.57E-06 | -0.03 | 0.02 | 0.12 | 0.01 | 21.09 |

Abbreviations: IV: instrumental variable; SNP: single nucleotide polymorphism; Chr: chromosome; Pos: position according to GRCh37/hg19 genome assembly; EA: effect allele; OA: other allele; EAF: effect allele frequency; SE: standard error of beta.

Table S3. MR estimates of assessing the causal effects of cystatin on lung cancer

| **Exposure** | **Outcome** | **Number of SNPs** | **Methods** | **OR (95% CI)** | **P** |
| --- | --- | --- | --- | --- | --- |
| Cystatin 8 | Lung adenocarcinoma | 20 | Inverse variance weighted | 1.041 (0.999-1.084) | 0.053 |
| Cystatin 8 | Lung adenocarcinoma | 20 | MR Egger | 1.041 (0.982-1.104) | 0.194 |
| Cystatin 8 | Lung adenocarcinoma | 20 | Simple mode | 1.122 (0.996-1.264) | 0.073 |
| Cystatin 8 | Lung adenocarcinoma | 20 | Weighted median | 1.042 (0.99-1.096) | 0.113 |
| Cystatin 8 | Lung adenocarcinoma | 20 | Weighted mode | 1.047 (0.992-1.105) | 0.115 |
| Cystatin 8 | Non-small cell lung cancer | 20 | Inverse variance weighted | 0.983 (0.9-1.075) | 0.712 |
| Cystatin 8 | Non-small cell lung cancer | 20 | MR Egger | 1.003 (0.881-1.144) | 0.959 |
| Cystatin 8 | Non-small cell lung cancer | 20 | Simple mode | 1.087 (0.864-1.368) | 0.486 |
| Cystatin 8 | Non-small cell lung cancer | 20 | Weighted median | 1.006 (0.899-1.125) | 0.916 |
| Cystatin 8 | Non-small cell lung cancer | 20 | Weighted mode | 1.014 (0.901-1.142) | 0.816 |
| Cystatin 8 | Squamous cell lung carcinoma | 19 | Inverse variance weighted | 1.062 (1.004-1.124) | 0.035 |
| Cystatin 8 | Squamous cell lung carcinoma | 19 | MR Egger | 1.083 (0.998-1.177) | 0.074 |
| Cystatin 8 | Squamous cell lung carcinoma | 19 | Simple mode | 1.112 (0.969-1.276) | 0.15 |
| Cystatin 8 | Squamous cell lung carcinoma | 19 | Weighted median | 1.064 (1.003-1.128) | 0.04 |
| Cystatin 8 | Squamous cell lung carcinoma | 19 | Weighted mode | 1.067 (1.006-1.131) | 0.044 |
| Cystatin B (ebi-a-GCST90085722) | Lung adenocarcinoma | 3 | Inverse variance weighted | 0.958 (0.815-1.126) | 0.605 |
| Cystatin B (ebi-a-GCST90085722) | Lung adenocarcinoma | 3 | MR Egger | 1.155 (0.667-2) | 0.698 |
| Cystatin B (ebi-a-GCST90085722) | Lung adenocarcinoma | 3 | Simple mode | 0.838 (0.641-1.096) | 0.326 |
| Cystatin B (ebi-a-GCST90085722) | Lung adenocarcinoma | 3 | Weighted median | 0.963 (0.804-1.154) | 0.681 |
| Cystatin B (ebi-a-GCST90085722) | Lung adenocarcinoma | 3 | Weighted mode | 0.991 (0.807-1.216) | 0.939 |
| Cystatin B (ebi-a-GCST90085722) | Squamous cell lung carcinoma | 2 | Inverse variance weighted | 1.023 (0.621-1.684) | 0.93 |
| Cystatin B (ebi-a-GCST90085722) | Non-small cell lung cancer | 3 | Inverse variance weighted | 1.121 (0.797-1.578) | 0.512 |
| Cystatin B (ebi-a-GCST90085722) | Non-small cell lung cancer | 3 | MR Egger | 1.327 (0.445-3.957) | 0.701 |
| Cystatin B (ebi-a-GCST90085722) | Non-small cell lung cancer | 3 | Simple mode | 1.061 (0.69-1.631) | 0.812 |
| Cystatin B (ebi-a-GCST90085722) | Non-small cell lung cancer | 3 | Weighted median | 1.125 (0.76-1.665) | 0.555 |
| Cystatin B (ebi-a-GCST90085722) | Non-small cell lung cancer | 3 | Weighted mode | 1.136 (0.765-1.689) | 0.592 |
| Cystatin B (prot-b-3) | Lung adenocarcinoma | 3 | Inverse variance weighted | 0.975 (0.895-1.063) | 0.568 |
| Cystatin B (prot-b-3) | Lung adenocarcinoma | 3 | MR Egger | 0.999 (0.775-1.288) | 0.997 |
| Cystatin B (prot-b-3) | Lung adenocarcinoma | 3 | Simple mode | 0.996 (0.93-1.067) | 0.923 |
| Cystatin B (prot-b-3) | Lung adenocarcinoma | 3 | Weighted median | 0.992 (0.932-1.056) | 0.798 |
| Cystatin B (prot-b-3) | Lung adenocarcinoma | 3 | Weighted mode | 0.995 (0.93-1.063) | 0.887 |
| Cystatin B (prot-b-3) | Non-small cell lung cancer | 4 | Inverse variance weighted | 0.963 (0.864-1.073) | 0.494 |
| Cystatin B (prot-b-3) | Non-small cell lung cancer | 4 | MR Egger | 1.061 (0.882-1.277) | 0.596 |
| Cystatin B (prot-b-3) | Non-small cell lung cancer | 4 | Simple mode | 1.006 (0.85-1.19) | 0.949 |
| Cystatin B (prot-b-3) | Non-small cell lung cancer | 4 | Weighted median | 0.976 (0.861-1.108) | 0.711 |
| Cystatin B (prot-b-3) | Non-small cell lung cancer | 4 | Weighted mode | 0.984 (0.864-1.12) | 0.824 |
| Cystatin B (prot-b-3) | Squamous cell lung carcinoma | 3 | Inverse variance weighted | 1.04 (0.956-1.131) | 0.366 |
| Cystatin B (prot-b-3) | Squamous cell lung carcinoma | 3 | MR Egger | 1.065 (0.954-1.189) | 0.465 |
| Cystatin B (prot-b-3) | Squamous cell lung carcinoma | 3 | Simple mode | 1.041 (0.915-1.184) | 0.605 |
| Cystatin B (prot-b-3) | Squamous cell lung carcinoma | 3 | Weighted median | 1.046 (0.952-1.149) | 0.346 |
| Cystatin B (prot-b-3) | Squamous cell lung carcinoma | 3 | Weighted mode | 1.052 (0.954-1.16) | 0.418 |
| Cystatin D | Lung adenocarcinoma | 10 | Inverse variance weighted | 1.013 (0.85-1.208) | 0.884 |
| Cystatin D | Lung adenocarcinoma | 10 | MR Egger | 0.924 (0.636-1.344) | 0.691 |
| Cystatin D | Lung adenocarcinoma | 10 | Simple mode | 1.209 (0.825-1.771) | 0.356 |
| Cystatin D | Lung adenocarcinoma | 10 | Weighted median | 1.039 (0.893-1.208) | 0.621 |
| Cystatin D | Lung adenocarcinoma | 10 | Weighted mode | 1.133 (0.835-1.539) | 0.443 |
| Cystatin D | Non-small cell lung cancer | 9 | Inverse variance weighted | 0.856 (0.651-1.127) | 0.269 |
| Cystatin D | Non-small cell lung cancer | 9 | MR Egger | 1.024 (0.562-1.867) | 0.939 |
| Cystatin D | Non-small cell lung cancer | 9 | Simple mode | 1.145 (0.7-1.873) | 0.603 |
| Cystatin D | Non-small cell lung cancer | 9 | Weighted median | 0.946 (0.699-1.28) | 0.718 |
| Cystatin D | Non-small cell lung cancer | 9 | Weighted mode | 1.209 (0.722-2.024) | 0.491 |
| Cystatin D | Squamous cell lung carcinoma | 9 | Inverse variance weighted | 1.025 (0.916-1.146) | 0.67 |
| Cystatin D | Squamous cell lung carcinoma | 9 | MR Egger | 1.098 (0.878-1.372) | 0.441 |
| Cystatin D | Squamous cell lung carcinoma | 9 | Simple mode | 1.122 (0.866-1.453) | 0.409 |
| Cystatin D | Squamous cell lung carcinoma | 9 | Weighted median | 1.055 (0.907-1.227) | 0.486 |
| Cystatin D | Squamous cell lung carcinoma | 9 | Weighted mode | 1.098 (0.862-1.399) | 0.47 |
| Cystatin F | Lung adenocarcinoma | 13 | Inverse variance weighted | 1 (0.963-1.038) | 0.984 |
| Cystatin F | Lung adenocarcinoma | 13 | MR Egger | 1.026 (0.973-1.082) | 0.364 |
| Cystatin F | Lung adenocarcinoma | 13 | Simple mode | 1.007 (0.925-1.097) | 0.871 |
| Cystatin F | Lung adenocarcinoma | 13 | Weighted median | 1.015 (0.971-1.06) | 0.511 |
| Cystatin F | Lung adenocarcinoma | 13 | Weighted mode | 1.016 (0.977-1.055) | 0.444 |
| Cystatin F | Non-small cell lung cancer | 13 | Inverse variance weighted | 1.007 (0.876-1.158) | 0.923 |
| Cystatin F | Non-small cell lung cancer | 13 | MR Egger | 1.022 (0.81-1.289) | 0.86 |
| Cystatin F | Non-small cell lung cancer | 13 | Simple mode | 0.841 (0.646-1.093) | 0.22 |
| Cystatin F | Non-small cell lung cancer | 13 | Weighted median | 0.95 (0.83-1.087) | 0.455 |
| Cystatin F | Non-small cell lung cancer | 13 | Weighted mode | 0.935 (0.803-1.089) | 0.403 |
| Cystatin F | Squamous cell lung carcinoma | 13 | Inverse variance weighted | 1.009 (0.966-1.054) | 0.688 |
| Cystatin F | Squamous cell lung carcinoma | 13 | MR Egger | 1.022 (0.958-1.091) | 0.526 |
| Cystatin F | Squamous cell lung carcinoma | 13 | Simple mode | 0.933 (0.828-1.053) | 0.283 |
| Cystatin F | Squamous cell lung carcinoma | 13 | Weighted median | 1.022 (0.969-1.078) | 0.429 |
| Cystatin F | Squamous cell lung carcinoma | 13 | Weighted mode | 1.018 (0.969-1.07) | 0.493 |
| Cystatin M (prot-a-703) | Lung adenocarcinoma | 9 | Inverse variance weighted | 0.95 (0.865-1.042) | 0.274 |
| Cystatin M (prot-a-703) | Lung adenocarcinoma | 9 | MR Egger | 0.988 (0.832-1.173) | 0.892 |
| Cystatin M (prot-a-703) | Lung adenocarcinoma | 9 | Simple mode | 0.935 (0.786-1.112) | 0.468 |
| Cystatin M (prot-a-703) | Lung adenocarcinoma | 9 | Weighted median | 0.947 (0.84-1.068) | 0.379 |
| Cystatin M (prot-a-703) | Lung adenocarcinoma | 9 | Weighted mode | 0.948 (0.806-1.116) | 0.54 |
| Cystatin M (prot-a-703) | Non-small cell lung cancer | 10 | Inverse variance weighted | 1.082 (0.863-1.357) | 0.496 |
| Cystatin M (prot-a-703) | Non-small cell lung cancer | 10 | MR Egger | 0.975 (0.618-1.538) | 0.916 |
| Cystatin M (prot-a-703) | Non-small cell lung cancer | 10 | Simple mode | 0.99 (0.668-1.469) | 0.962 |
| Cystatin M (prot-a-703) | Non-small cell lung cancer | 10 | Weighted median | 1.117 (0.865-1.442) | 0.396 |
| Cystatin M (prot-a-703) | Non-small cell lung cancer | 10 | Weighted mode | 1.08 (0.796-1.465) | 0.632 |
| Cystatin M (prot-a-703) | Squamous cell lung carcinoma | 10 | Inverse variance weighted | 0.999 (0.842-1.184) | 0.987 |
| Cystatin M (prot-a-703) | Squamous cell lung carcinoma | 10 | MR Egger | 0.983 (0.69-1.401) | 0.928 |
| Cystatin M (prot-a-703) | Squamous cell lung carcinoma | 10 | Simple mode | 1.016 (0.807-1.278) | 0.898 |
| Cystatin M (prot-a-703) | Squamous cell lung carcinoma | 10 | Weighted median | 1.044 (0.899-1.213) | 0.572 |
| Cystatin M (prot-a-703) | Squamous cell lung carcinoma | 10 | Weighted mode | 1.043 (0.873-1.246) | 0.654 |
| Cystatin M (prot-a-704) | Lung adenocarcinoma | 13 | Inverse variance weighted | 0.956 (0.887-1.03) | 0.235 |
| Cystatin M (prot-a-704) | Lung adenocarcinoma | 13 | MR Egger | 0.986 (0.844-1.151) | 0.858 |
| Cystatin M (prot-a-704) | Lung adenocarcinoma | 13 | Simple mode | 0.932 (0.785-1.108) | 0.44 |
| Cystatin M (prot-a-704) | Lung adenocarcinoma | 13 | Weighted median | 0.979 (0.885-1.083) | 0.677 |
| Cystatin M (prot-a-704) | Lung adenocarcinoma | 13 | Weighted mode | 1.012 (0.88-1.164) | 0.868 |
| Cystatin M (prot-a-704) | Non-small cell lung cancer | 14 | Inverse variance weighted | 0.98 (0.833-1.154) | 0.813 |
| Cystatin M (prot-a-704) | Non-small cell lung cancer | 14 | MR Egger | 1.051 (0.748-1.479) | 0.778 |
| Cystatin M (prot-a-704) | Non-small cell lung cancer | 14 | Simple mode | 1.02 (0.707-1.473) | 0.917 |
| Cystatin M (prot-a-704) | Non-small cell lung cancer | 14 | Weighted median | 1.025 (0.82-1.282) | 0.828 |
| Cystatin M (prot-a-704) | Non-small cell lung cancer | 14 | Weighted mode | 1.124 (0.829-1.522) | 0.466 |
| Cystatin M (prot-a-704) | Squamous cell lung carcinoma | 12 | Inverse variance weighted | 1.04 (0.936-1.156) | 0.463 |
| Cystatin M (prot-a-704) | Squamous cell lung carcinoma | 12 | MR Egger | 1.137 (0.916-1.41) | 0.272 |
| Cystatin M (prot-a-704) | Squamous cell lung carcinoma | 12 | Simple mode | 1.04 (0.842-1.286) | 0.721 |
| Cystatin M (prot-a-704) | Squamous cell lung carcinoma | 12 | Weighted median | 1.056 (0.929-1.2) | 0.404 |
| Cystatin M (prot-a-704) | Squamous cell lung carcinoma | 12 | Weighted mode | 1.043 (0.882-1.234) | 0.632 |

Table S4. The causal association between cystatin levels and lung cancer after outliers removal

| Exposure | Outcome | N.SNP | Method | OR (95% CI) | P | |
| --- | --- | --- | --- | --- | --- | --- |
| Cystatin D plasma levels | Lung adenocarcinoma | 7 | Inverse variance weighted | 1.178 (1.023 , 1.358) | | 0.023 |
| Cystatin D plasma levels | Lung adenocarcinoma | 7 | MR Egger | 1.298 (0.947 , 1.781) | | 0.166 |
| Cystatin D plasma levels | Lung adenocarcinoma | 7 | Weighted median | 1.171 (1.007 , 1.362) | | 0.04 |
| Cystatin D plasma levels | Lung adenocarcinoma | 7 | Weighted mode | 1.183 (0.949 , 1.474) | | 0.185 |
| Cystatin M plasma levels (prot-a-703) | Squamous cell lung carcinoma | 9 | Inverse variance weighted | 1.069 (0.949 , 1.206) | | 0.272 |
| Cystatin M plasma levels (prot-a-703) | Squamous cell lung carcinoma | 9 | MR Egger | 0.944 (0.764 , 1.168) | | 0.613 |
| Cystatin M plasma levels (prot-a-703) | Squamous cell lung carcinoma | 9 | Weighted median | 1.063 (0.909 , 1.242) | | 0.445 |
| Cystatin M plasma levels (prot-a-703) | Squamous cell lung carcinoma | 9 | Weighted mode | 1.035 (0.85 , 1.259) | | 0.742 |
| Cystatin F plasma levels | Non-small cell lung cancer (all cancers excluded) | 11 | Inverse variance weighted | 0.940 (0.8433 - 1.0479) | | 0.264 |
| Cystatin F plasma levels | Non-small cell lung cancer (all cancers excluded) | 11 | MR Egger | 0.9753 (0.8285 - 1.1481) | | 0.771 |
| Cystatin F plasma levels | Non-small cell lung cancer (all cancers excluded) | 11 | Weighted median | 0.9403 (0.8201 - 1.0781) | | 0.378 |
| Cystatin F plasma levels | Non-small cell lung cancer (all cancers excluded) | 11 | Weighted mode | 0.9409 (0.8193 - 1.0806) | | 0.409 |

Table S5. The heterogeneity and pleiotropy assessment for the causal association between cystatin levels and lung cancer after outliers removal

| **Exposure** | **Outcome** | **Heterogeneity** | | **Pleiotropy** | |
| --- | --- | --- | --- | --- | --- |
|  |  | **Q statistic (IVW)** | ***P* value** | **MR-Egger Intercept** | ***P* value** |
| Cystatin D plasma levels | Lung adenocarcinoma | 10.052 | 0.122 | -0.01876 | 0.526 |
| Cystatin M plasma levels (prot-a-703) | Squamous cell lung carcinoma | 10.134 | 0.256 | 0.03808 | 0.216 |
| Cystatin F plasma levels | Non-small cell lung cancer | 6.868 | 0.738 | -0.00509 | 0.878 |

Table S6. The identification of outliers in the association between cystatin F levels and NSCLC by Radial MR analysis

| **rsid** | **Outliers_IVW** | **Outliers_Egger** |
| --- | --- | --- |
| rs111825697 | Variant | Variant |
| rs113656409 | Variant | Variant |
| rs143377863 | Outlier | Outlier |
| rs183113549 | Variant | Variant |
| rs186258664 | Variant | Variant |
| rs2387362 | Variant | Variant |
| rs4894484 | Variant | Variant |
| rs57832775 | Variant | Variant |
| rs6577309 | Variant | Variant |
| rs6812091 | Variant | Variant |
| rs6993770 | Variant | Variant |
| rs7269564 | Outlier | Outlier |
| rs7297614 | Variant | Variant |
